# Impact of *Chlorella vulgaris*, *Nannochloropsis salina*, and *Arthrospira platensis* as bio-stimulants on common bean plant growth, yield and antioxidant capacity

**DOI:** 10.1038/s41598-023-50040-4

**Published:** 2024-01-16

**Authors:** Fatma Abd El Lateef Gharib, kholoud Osama, Amira Mohamed Abd El Sattar, Eman Zakaria Ahmed

**Affiliations:** Botany and Microbiology Department, Faculty of Science, Helwan University, Helwan, Egypt

**Keywords:** Plant sciences, Ecology, Environmental sciences

## Abstract

The use of bio-stimulants in agriculture has emerged as a promising strategy to improve crop growth and yield. One type of bio-stimulant that has gained attention is microalgae extracts, which are known for their high metabolic activity, bioactive compounds, and ability to enhance plant growth and development. To investigate their effectiveness, a pot experiment was conducted at the Experimental Farm of Helwan University in Egypt during the 2022 season. The experiment aimed to evaluate the efficacy of *Chlorella vulgaris*, *Nannochloropsis salina*, and *Arthrospira platensis (Spirulina platensis)* extracts as bio-stimulants, applied through foliar spray at concentrations ranging from 0.25 to 2.0%, on common bean plants. Analysis of algal extract showed that .* N. salina* had the highest content of promotive growth hormones gibberellins (GA_3_) (74.85 ± 2.7mg100 g^−1^ d.wt). and auxins (IAA) (34.57 ± 2.7µg 100 g^−1^ d.wt.) compared to *Chlorella* and *Arthrospira*..The results revealed that the application of *C. vulgaris*, *N. salina*, and *A. platensis* extracts at concentrations up to 1.0% significantly improved various growth parameters, such as root, and shoot length, number of leaves and flowers per plant, leaf area, and total fresh and dry weight per plant. These extracts also positively affected yield attributes, including the number and fresh weight of pods per plant, seed index, seed yield per plant, and per feddan [a unit of land area]. Furthermore, the application of these extracts increased the chlorophyll content index with the maximum values of CCI (17.95. and 17.81%) was obtained at 0.50% *N. salina*, followed by 0.50% *C.vulgaris.* In addition to increase in the capacity of both non-enzymatic antioxidants [such as total antioxidant capacity, phenolics, and flavonoids] and enzymatic antioxidants [including catalase and ascorbic oxidase]. The most promising results were observed with the application of *N. salina,* and *C. vulgaris* extracts at a concentration of 0.5%. Additionally, the extracts significantly reduced the content of oxidative stress markers, such as malondialdehyde, percentage of electrolyte leakage, and hydrogen peroxide, in common bean plants compared to the control group. Contrarily, the measured parameters were reduced, while the levels of oxidative stress markers and some antioxidants including peroxidase, ascorbic peroxidase, superoxide dismutase, glutathione peroxidase, and glutathione transferase were increased by three algal extracts at a concentration of 2.0%, compared to control plants. Additionally, the application of these microalgae extracts improved the quality parameters, proximate composition, seed energy, and mineral contents of the harvested seeds, with the most significant positive impact was observed at 0.5% concentration of algal extract. These findings demonstrate the successful and safe utilization of extracts from *C. vulgaris*, *N. salina*, and *A. platensis* at concentrations up to 1.0% as bio-stimulants to enhance common bean yields and improve the nutritional quality of dried beans for consumers.

## Introduction

Common bean [Family *Fabaceae*] is a herbaceous vegetable crop consumed worldwide^1^ and one of the most important and well-known leguminous crops used for human nutrition, with a commercial value exceeding that of all other bean crops^2^. It is the second-most important source of human dietary proteins and the third-most important source of calories^3^. It provides a valuable source of complex carbohydrates, dietary fiber, and phytocompounds with analgesic and neuroprotective properties^4^. Common bean [*Phaseolus vulgaris L*.] constitutes an integral portion of the diet of many people in rural and poor urban communities and meets more than 50% of the diet protein supplies of households in Africa^5^.

Despite their low content of Sulfur amino acids, dry beans seeds are a major source of nutritional low fat protein, containing 21 to 25% protein by weight, which is 2–3 times higher than that found in cereal grains^6^. Dry beans are rich in nutrients that have been proven to protect against various diseases, including cardiovascular disease, obesity, diabetes, metabolic syndrome, and cancer^7^. The polyphenols present in dry beans have antioxidant properties and can prevent the formation of free radicals^1^. Due to their nutritional composition, regular consumption of common beans has the potential to improve diet quality and long-term health^8^. However, despite their importance for nutrition and the economy, common beans have low yields and may struggle to meet the food demands of growing populations.

Common bean cultivation is often challenged by low adoption of improved technologies, poor agronomic practices, environmental degradation, and stresses^9^, resulting in low yield and quality that cannot meet food demands of growing populations. Bio-stimulants have emerged as alternatives to chemical inputs, and promising approach to enhance crop growth, yield and, quality with less environmental damage^10^.

Among bio-stimulants, *Chlorella*, *Nannochloropsis,* and *Arthrospira* have recognized as a valuable biofertilizers rich in different bioactive compounds, minerals and organic nutrients. ***Chlorella**** vulgaris* (Cholorophyta family) is green unicellular microalgae. It contains 45.23% protein, 23.43% carbohydrate, 18.12% total lipids, 10% minerals and vitamins, 5% fiber, phenolic compounds, chlorophyll a, b pigments, and carotenoids^11,12^. The predominant fatty acids are linolenic, linoleic, and palmitic along with high concentrations of glutamine^13^. Additionally, this microalga has brassinosteroids and gibberellin hormones^14^. Recently, *Nannochloropsis* has expected increasing interest in research. *Nannochloropsis* sp. [Eustigmatophyceae family] is a genus of fast-growing unicellular, marine green microalgae. *Nannochloropsis* contains 36% of the biomass protein^15^**,** 25 to 35% of their dry weight lipid, including triacylglycerol and the omega-3 [ω3] long chain polyunsaturated fatty acid in the form of eicosapentaenoic acid^16,17^, along with carbohydrates, vitamins, bio-active acids, and microelements. The pigments composition of *Nannochloropsis* sp are chlorophyll *a*, violaxanthin and carotenoids, such as lutein and β-carotene, which play an important role in antioxidant activities^18^. On the other hand, *Arthrospira* sp. is blue-green algae. *Arthrospira platensis* (Cyanobacterial family) has commercial importance for plants as a source of nutrients, proteins**,** carbohydrates, minerals, vitamins, essential amino acids, fatty acids, polypeptides, phytohormones, and antioxidant compounds^19^. These algae could be a new option of bio-stimulants, and bio-fertilizers for organic cultivation of plants.

Recently, studies showed microalgae-based bio-stimulants as a promising, environmentally friendly, and sustainable agricultural technique for increased crop yield and sustainability^20^. For example, *Arthrospira platensis* increased dry weight of root and shoots, number of leaves, and flowers of *Petunia* x *hybrid*^21^, and. show cytokine-like effects on lettuce seedlings^22^. *Chlorella vulgaris* suspension stimulate cucumber, and tomato seeds germination^23^, and positively affected the initial growth of Swiss chard, and the pigments of photosynthsis at 5% suspensions^24^. In addition, *C. vulgaris*, *N. salina* and* S. platensis* have pivotal role in building and sustaining soil fertility, thereby raising the biomass, quality, and yield of black gram [*Vigna mungo* L.]^25^, onion [*Allium cepa* L.]^26^, and *Moringa oleifera* under salinity stress^27^.

Based on the above information, the current study aimed to evaluate the efficacy of *Chlorella vulgaris*, *Nannochloropsis salina*, and *Arthrospira platensis* extracts as bio-stimulants on growth and productivity of Bronco variety of common bean (*Phaseolus vulgaris* L.) plants, including CCI, oxidative stress marker, antioxidant activity, yield, and yield performance. Additionally, we assessed the impact of different strains of algae on the quality and nutritional values (proximate compositions, energy, and minerals) of the yielded seeds.

## Materials and methods

### Materials

A uniform batch of seeds of the Bronco variety of common bean [*Phaseolus vulgaris* L.] was supplied by the Horticulture Research Institute, Agriculture Research Center, Ministry of Agriculture, Giza, Egypt.

Dry *Chlorella vulgaris,* *Nannochloropsis salina* and *Arthrospira platensis* algae were obtained from the Algal Biotechnology Unit, National Research Centre, Dokki, Egypt.

### Preparation of algal extracts

The extraction of air-dried powder from *Chlorella vulgaris*, *Nannochloropsis salina* and *Arthrospira platensis* algae was performed using 80% methanol [PIOCHEM], following the method described by^28^. The solvent was evaporated under reduced pressure at 40 °C using a rotatory evaporator. The resulting dried extracts were dissolved in distilled water in a ratio of w [residue] to v [distilled water] to achieve the desired concentration.

### Experimental design

In the autumn season of 2022, a pot experiment was done at Helwan University's Experimental Farm in Cairo. *Phaseolus vulgaris* L. seeds were sown on August 21. Each earthen-ware pot, measuring 30 cm in depth, and 25 cm in diameter contained 10 kg of soil and five seeds were sown per pot. The soil selected for the experiment was clay loamy soil with clay content of 54.96%, silt content of 26.04%, fine sand content of 11.14%, and coarse sand content of 7.86%. Clay loamy soil is characterized by suitable water holding capacity and good aeration.

After one week of growth, pots were divided into four groups. Except the negative control group, the other three groups were further subdivided into either 4 sub-groups, with each sub-group consisting of 10 pots with three plants in each replicate a total of 30 plants. These pots were then treated with three different algal extract solutions. The pots were set up in a completely randomized block arrangement with thirteen treatments including three algal extracts (*C. vulgaris,* *S. platensis* and *N. salina*) at four concentrations (0.25, 0.50, 1.0 and 2.0%).

The irrigation schedule was determined based on the weather conditions and the goal was to maintain the soil moisture at field capacity. This means that the soil should have enough moisture to support plant growth without becoming waterlogged. Other conventional agricultural practices were followed for bean planting.

Fertilization was done using three types of fertilizers: ammonium nitrate (33.5% nitrogen content), P_2_O_5_ (15.5% phosphorous content), and K_2_SO_4_ (48% potassium content) as normal soil fertilizers added to kidney bean plants in field. Each pot received 1 g of each fertilizer. Fertilizer was used.in two equal doses. The first dose was added at the time of sowing. After 30 days, the second dose was applied.

During the vegetative growth stage of the Bronco plants, a foliar spray was administered twice at 22 and 29 Day after sowing (DAS). The control group received a foliar spray using dist. water. The spraying solution was applied in a volume that ensured full coverage of the plant foliage until it began to drip.

Plant growth characters measured at flowing stage (40 DAS): twelve plants (6 replicates) were drawn from each treatment and plant growth characteristics were measured including plant height (cm) (PH), root length (RL), leaf number plant^−1^ (LN), flower number plant^−1^ (FN), leaf area (cm^2^ plant^−1^) (LA) according to Koller^29^, fresh (FW) and dry weight (DW) (g plant^−1^). Total phenolic (TP) and flavonoids (TF) contents were determined in dry leaves. Representative fresh samples of leaves and roots were collected from different treatments to identify oxidative stress indicators and certain antioxidant activity.

At the time of harvest (i.e., 100 DAS): twelve plants were chosen randomly from each treatment, to estimate yield and yield attributes morphologically. The following characteristics of plant yield were recorded. These represented pod length (PL) (cm), number of pods plant^−1^ (PN P^−1^), number of seeds pod^−1^ (SN P^−1^), number of seeds per plant (SN Pl^−1^), pods weight plant^−1^ (g) (PW P^−1^), 100-seed weight (100-SW) (seed index) (SI), seed yield plant^−1^ (g) (SY P^−1^), and per yield (ton feddan^−1^) (SY F^−1^).

Harvested seeds from different groups were dried in an electric oven with a drift fan at 70 °C for 48 h and used for determination of proximate chemical composition [moisture, total fat, crude fiber, ash, crude protein, total carbohydrates, and energy], and mineral content.

### Ethical approval

Experimental research and field studies on plants comply with relevant institutional, national, and international guidelines and legislation.

## Chemical analysis

### Phytohormones analysis of algae

Air dry samples of the ***Chlorella vulgaris*, *Nanochloropsis salina*, and *Arthrospira platensis*** microalgal were extracted according to the method adopted by Wasfy and Orrin^30^. Extraction and detection of phytohormone (auxins (IAA), gibberellins (GA_3_), and abscisic acid (ABA)). were performed at Arid Land Research Center, Faculty of Agriculture, Ain Shams University. The identification and measurement of hormones were carried out by injecting of 10 µl into the HPLC 510 using the data model (waters 746), detector (U.V. Tumable Absorbance), and pump (HPLC 510).

### Chlorophyll content index

Chlorophyll content index [CCI] was measured in *P. vulgaris* leaves at 40 DAS with a Chlorophyll Meter [CCM-200; ADC Bioscientific, Hoddesdon, UK] by clipping the sensor onto the third fresh leaf from top [10 randomly selected leaves were measured for each treatment]. The absorbance was measured at two distinct wavelengths, specifically 653 nm and 931 nm, which fall within the Near Infra-Red range.

### Oxidative stress markers

#### Lipid peroxidation

Thiobarbituric acid [TBA] test was used to measure lipid peroxidation in leaf and root tissues according to Heath and Packer^31^. This test allows for the detection of malondialdehyde (MDA]), a byproduct of lipid peroxidation. Leaf and roots materials weighing 0.5 g each were homogenized in 10 ml of TBA reagent, which consisted of a mixture of 18% trichloroacetic acid and 0.45% TBA in a 1:2 ratio. After 15-min of incubation in a hot water bath, the mixture was filtered. Following filtration, the reaction was stopped by submerging the tubes in an ice bath.

Next, the samples were centrifuged for 10 min at a speed of 6000 revolutions per minute [rpm]. The absorbance of the supernatant was then measured at a wavelength of 532 nm using a spectrophotometer. Non-specific absorbance measured at 600 nm was subtracted from absorbance at 532 nm. Finally, MDA quantity was measured in units of µmol g^-1^ FW. equivalent.

#### Membrane permeability

The permeability of cell membranes was measured using electrolyte leakage according to Zwiazek and Blake^32^. After being thoroughly cleaned, 2.5 g fresh leaves and roots were segmented into 2-cm lengths and put into separate glass vials containing 25 mL of de-ionized water. The electrolytic conductivity [EC1] of the bathing solution was measured with a conductivity metre [Model Ohm-419] after 30 min of soaking in water. The roots, and leaves were then boiled, and the EC was measured once more [EC2] after cooling the bathing solution to room temperature. The relative permeability of the plasma membrane was calculated as follows:

$${\text{Relative}}\;{\text{ permeability}}\left[ \% \right] = \, \left[ {{\text{EC1}}/{\text{EC2}}} \right] \, \times { 1}00$$

#### Hydrogen peroxide content

According to Velikova et al.^33^ method, the level of H_2_O_2_ was measured in fresh tissue. In an ice bath, 0.5 g of leaf tissue was homogenised in 5 mL of 0.1% (w/v) trichloroacetic acid. Centrifuging the homogenate at 6000 rpm and 4 °C for 30 min. To the supernatant [1.0 M], 1.0 mL KI and 0.5 mL K_3_PO_4_ buffer (10 mM, pH 7.0) were added. The absorbance of the supernatant was determined at 390 nm. For the blank solution, 0.5 mL distilled H_2_O was substituted for of plant extract buffer. On a standard curve, the H_2_O_2_ concentration was determined, and the findings were represented as µmol g^−1^ FW. equivalent.

### Non-enzymatic antioxidants

#### Total antioxidant capacity

The total antioxidant capacity of the fresh common bean leaf extract was measured by Kholssi^34^ method using a colorimetric assay kits, Biodiagnostic Co., Egypt. To perform the assay, 0.02 mL of the extract solution with a concentration of 1 mg mL^−1^ was added to 0.05 mL R1 substrate (H_2_O_2_) and incubated at 37 °C for 10 min. Then, 0.5 mL of the working reagent, which contained equivalent volumes of Chromogen (R2) and enzyme-buffer (R3), was added to the mixture. The reaction was mixed and further incubated at 37 °C for 5 min. A blank solution was prepared with 0.02 mL of dist. H_2_O in place of the enzyme extract solution. Then, at 505 nm, the absorbance of the sample and blank solutions was evaluated against distilled water. The antioxidant capacity was calculated and expressed as mM L^−1^ of the extract**.**

#### Total phenolic content

The air-dried powdered leaves of common bean [0.1 g] were extracted at room temperature by agitating 25 mL of 70% ethanol at room temperature until the extraction solvent turned clear. Using the Folin-Ciocalteu reagent and gallic acid as a standard, the total phenolic content of the extract was assessed, following the method described by Kujala et al.^35^, 0.5 mL of the filtered extracts were mixed with 2.5 mL of diluted Folin-Ciocalteu reagent [1:10 ethanol] and 2 mL Na_2_CO_3_ (7.5%). The mixture was thoroughly mixed and then incubated at room temperature for 15 min. Using a Jenway 6405 UV–Vis spectrophotometer and a blank reagent, the absorbance of the resulting blue-colored solution was determined at 765 nm. The total phenolic content of the extract was calculated as mg of gallic acid equivalents (GAE) per gram of extract (mg GAE g^−1^ DW.).

#### Total flavonoids

The amount of total flavonoid in the common bean extract was determined using AlCl_3_ colorimetric assay according to Piyanete et al.^36^. Sample solutions consisting of 0.1 g air dry leaves in 25 mL of 70% ethanol were prepared. Then, 0.5 mL of sample solutions was added to 2 mL dist.H_2_O and 0.15 mL of a 5% NaNO_2_ solution. After incubating for six minutes, 0.15 mL of a 10% AlCl_3_ solution was added and left for another six minutes. Subsequently, 2 mL of a 4% NaOH solution was added. The mixture was then well mixed and diluted with methanol to 5 mL. The absorbance was measured using a spectrophotometer at 510 nm after a 15-min incubation period, against a blank. The total flavonoid content was quantified as mg of quercetin equivalents [QE] per gram of dry weight [mg QE g^−1^ DW.]. To calculate the total flavonoids concentration, a quercetin standard curve was utilized.

### Antioxidants enzymes

#### Ascorbic acid oxidase [EC 1.10.3.3]

Ascorbic acid oxidase (AOX) was assayed by Farkas and Kiraly^37^ and modified version of Maxwell and Bateman^38^ technique. The reaction mixture in a quartz cuvette contained 1.0 mL of 0.2 M phosphate buffer [pH 6.2], 0.2 mL of 1 mM ascorbic acid, 0.2 mL of crude enzyme completed to 3.0 mL with dist. H_2_O. The initial absorbance [A_0_] recorded immediately at 265 nm. Then, the rate of disappearance of ascorbate was followed by reading optical density after 30 s interval up to 3 min at 265 nm. Activity of ascorbic acid oxidase expressed as g^−1^ fresh weight equivalent hour^−1^.

#### Ascorbate peroxidase [EC 1.11.1.11]

Ascorbate peroxidase (APX) enzyme was extracted from 0.5 g fresh leaf with 5 mL Tris–HCl buffer 50 mmol L^−1^ [pH 7.8] as the method described by Rama-Devi and Prasad^39^. After centrifugation for 20 min at 10.000 × g [12.000 rpm] and 40 °C, the supernatant was utilised to estimate APX in accordance with Nakano and Asada^40^.

The reaction mixture contained, enzymatic extract, 50 mmol L^−1^ cold sodium phosphate buffer [pH 7], 0.5 mmol L^−1^ ascorbate, 0.1 mmol L^−1^ H_2_O_2_, and 0.1 mmol L^−1^ EDTA, in a 3.0 mL final volume. The reaction started after the H_2_O_2_ addition, and the absorbance was determined spectrophotometry at 40 s interval and 290 nm. The molar extinction coefficient 2.8 mmol^−1^ cm^−1^ was used to calculate ascorbate peroxidase activity. Ascorbate peroxidase was expressed as g^−1^ fresh weight hour^−1^.

#### Peroxidase [EC 1.11.1.7]

Peroxidase was assayed in fresh common bean leaf following modified version of Yamane et al.^41^. Assay mixture contains 2.2 mL of 0.1 M K_3_PO_4_ buffer [pH 6.0], 0.5 mL of guaicol [0.018 mM], 0.2 mL H_2_O_2_ [30%], and 0.1 mL enzyme extract. The intensity of the colour was measured at 436 nm by recording changes in absorbance each 30 s up to 3 min. Optical density g^-1^ FW. hour^−1^ was used to express enzyme activity.

#### Superoxide dismutase [EC 1.15.1.1]

The activity of superoxide dismutase [SOD] enzyme in fresh leaf was conducted following the method outlined by Nishikimi et al.^42^. A commercially available SOD Biodiagnostic ready kit from Biodiagnostic Co. was utilized for the assay. For extraction, 0.25 g leaf tissue was homogenized in 5 mL cold potassium buffer [pH 7.0] with a concentration of 100 mM, followed by centrifugation at 4000 rpm and 4 °C for 15 min. Next, 0.5 mL of absolute cold ethanol/chloroform mixture [60/40, v/v] was added to 1.0 mL of the supernatant in a glass tube. The mixture was thoroughly mixed for at least 30 s and then centrifuged again at 4000 rpm and 4 °C for 10 min. The resulting supernatant from the extraction process was used to perform the assay. To prepare the assay mixture, 2 mL of 50 mM K_3_PO_4_ buffer with a pH of 8.5, 1 mM nitrobluetetazolium, and 1 mM NADH were blended together in a 10:1:1 ratio. Then, 0.2 mL enzyme extract was added to the mixture, along with 0.2 mL of dist. H_2_O. For the control assay, the same mixture was used, but without the enzyme extract. This control mixture was also mixed and put into a clean quartz cuvette. To start the reaction, 0.2 mL of 0.1 mM phenazine methosulphate [PMS] was added into the cuvette, which had been diluted 1000 times before use. The solution was immediately mixed after adding the PMS. The cuvette was then inserted into a spectrophotometer at a temperature of 25 °C for 5 min. The spectrophotometer recorded the increase in absorbance at a wavelength of 560 nm for both the control (Δ control) and the leaf sample (Δ sample). The activity of SOD enzyme is expressed as units per gram of fresh tissue (U g^−1^).

#### Glutathione peroxidase [EC 1.11.1.9]

Glutathione peroxidase [GSH-Px] was assessed in a fresh leaf as mentioned by Paglia and Valentine^43^ using Kits (**CAT. No. GP 2524**), Biodiagnostic Co. The GSH-Px activity is expressed as U L^−1^. The oxidation of NADPH to NADP^+^ is accompanied by a reduction in absorbance at 340 nm [A_340_] providing a spectrophotometric means for monitoring GSH-Px enzyme activity. Dilute [R3] 100 times immediately before use [0.1 mL + 10 mL d. H_2_O]. Assaying mixture contains buffer [R1] 1.0 mL, NADPH [R2] 0.1 mL, crude enzyme 0.01 mL, and H_2_O_2_ [R3] 0.1 mL Mix well, and record the decrease of absorbance at 340 nm/ min. [A_340_/min] throughout a three minute period against deionized water.

#### Glutathione transferase

Glutathione transferase (GSH-T] was assayed spectrophotometrically in the fresh bean tissue as reported by Habig et al.^44^, using Kits (**CAT. No. GT 2519**), Biodiagnostic Co., Egypt. This was done by using reduced glutathione [GSH] and 1-chloro-2, 4-dinitrobenzene (CDNB) as substrates. Observing the rise in absorbance of sample [A _sample_] at 340 nm against the blank, every one minute, for 5 min. Assaying mixture contains 1 mL of buffer, 0.05 mL crude enzyme extract and 0.1 mL of GSH. To the blank cuvette added PBS instead of crude extract. After incubation at 37 °C for 5 min, add 0.1 mL CDNB to sample only, Mix well. Incubate at 37 °C for 5 min. Terminate the reaction with 0.1 mL of trichloroacetic acid, and add to blank only 0.1 mL CDNB. Mix well, centrifuge at 3000 rpm for 5 min. The activity of GSH-T is expressed as** U L**^**−1**^,

#### Catalase [EC 1.11.1.6]

Leaf of common bean was utilized to prepare enzyme extracts as conducted by Kar and Mishra^45^, The process involved homogenizing 0.5 g fresh leaf in 10.0 mL of cold phosphate buffer (Na/ K phosphate 0.1 M, pH 6.8). After centrifuging the mixture for 10 min at 6000 rpm and 4 °C, the supernatants were adjusted to a known volume and used for catalase (CAT) assay. The catalase activity was assessed using an altered version of the technique developed by Góth^46^. In the reaction, 1 ml of H_2_O_2_ (65 mM H_2_O_2_ in N/KP pH 7.4), and 0.2 ml of crude enzyme extract was added to the reaction. The reaction was allowed to incubate at 25 °C for 4 min before being stopped by adding 1 mL of ammonium molybedate (4 g L^−1^). The remaining H_2_O_2_ was measured by monitoring the decrease in absorbance at 405 nm. A control was also performed simultaneously, where the activity was halted immediately at zero time. The activity of catalase was expressed as µM H_2_O_2_ destroyed g^−1^ fresh weight equivalent hour^−1^.

### Determination of nutrients

The yield seeds of dry common beans from different treatments were ground into flour, and subsequently utilized to assess their nutrient content, encompassing proximate compositions, energy, and minerals.

#### Proximate compositions

The proximate compositions, including moisture content, ash, crude lipid [fat], crude fiber, crude protein, carbohydrate, and energy content, were determined using standard Methods of the Association of Official Analytical Chemists^47^ in Integrated Control Research, Plant Pathology Research Institute, ARC, Giza, Egypt. Samples were analyzed in triplicate. Briefly, moisture content was determined by drying samples in an electric oven at 105 ± 5 °C for 6–8 h till the weight remained constant (**AOAC Method No. 925.09**)*,* ash percentage was measured by ignition organic matters in muffle furnace at 550 ± 5 °C for 2 h until the sample was free of carbon, cooled in a desiccator, and calculated for the amount of ash (**AOAC Method No. 923.03**). Total nitrogen was determined by the Kjeldahl method (**AOAC Method No. 979.09**), and crude protein (CP) was calculated by multiplying the values of total N by 6.25, which correspond to a nitrogen to protein conversion factor. Crude fat (total lipids) was determined by solvent extraction with petroleum ether using Soxhlet apparatus (**AOAC Method No. 920.29**). Crude fiber was determined according to (**AOAC Method No. 978.10**) after digesting common bean seeds by refluxing boiling H_2_SO_4_ (1.25%), and boiling KOH((28%), then filtration and washing was performed, followed by drying at 130 °C, and combustion for 30 min at 550 °C in muffle furnace. The fiber was calculated as a residue after subtraction of the ash. The percentage of total carbohydrate was calculated by subtracting measured protein, fat, ash and moisture from 100%, using Eq. (1), while energy value was calculated by the Atwater factor using Eq. (2): based on the three groups of nutrients (carbohydrates, fats and proteins).

1 $${\text{Total }}\;{\text{carbohydrate}}\; \, \% \, = { 1}00 \, {-}{\text{ Sum }}\;{\text{of}}\;{\text{ percentage}}\;{\text{ of }}\;\left( {{\text{moisture }} + {\text{ total ash }} + {\text{ fat }} + {\text{ protein}}} \right).$$

2 $${\text{Energy}}\;{\text{content }}\left( {{\text{Kcal g}}^{{ - {1}}} } \right) \, = \, \left( {{\text{Total }}\;{\text{carbohydrate}} \times { 4}} \right) \, + \, \left( {{\text{protein }} \times { 4}} \right) \, + \, \left( {{\text{total }}\;{\text{fat }} \times {9}} \right)$$

#### Determination of minerals

Mineral ions content in air-dry seeds of common bean developed from different treatments were estimated using the Microwave Plasma Atomic Emission Spectroscopy (Agilent Technologies 4210 MP-AES) instrument at Ecology lab.—Faculty of Science, Helwan University. The instrument was adjusted as explained by the manufacture’s user manual to ensure accurate and precise measurements. The seeds samples were first dried at 110 °C for 24 h, then crushed and sieved via a 2 mm sieve. 0.5 g of the sieved sample was digested in a 15 mL acid mixture of HNO_3_: HCl (1:1, v/v). The mixture was heated on a hot plate until the digest became clear. After the digestion process, the mixture was cooled, filtered, and completed to 25 mL with twice-de-ionized H_2_O. The phosphorus, potassium, magnesium, and calcium concentrations were determined and expressed as ppm on dry matter base.

### Statistical analysis

In this study, the data for growth criteria were reported as the mean value ± standard error (SE) of 6 replicates, while the data for chemical analysis were reported as the mean value ± SE of three 3 replicates. To analyze the data statistically, a one-way analysis of variance (ANOVA) was performed. Duncan’s Multiple Comparison Test was then used to compare the means, using IBM Statistical Product and Service Solutions (SPSS) Statistics for Windows, Version 21. A significance level of *P* < 0.05 was considered statistically significant, and the least significant difference (LSD) at the 5% level was used to compare the means.

## Results

### Phytohormone composition of microalgae

The results presented in Table 1 shows that phytohormone content is species dependent.* N. salina* followed by *C. vulgaris* had the highest content of gibberellins (GA_3_) (74.85 ± 2.7 mg100 g^−1^ DW). and auxins (IAA) (34.57 ± 2.7 µg 100 g^−1^ DW.), while *A. platensis* had the lowest content of GA_3_ (49.44 ± 6.50 mg100 g^−1^ DW). and similar level of IAA (26.29 ± 2.7 µg 100 g^−1^ DW.), to *C. vulgaris* (26.76 ± 2.7 µg 100 g^−1^ DW.). However, abscisic acid (ABA) was not detected in three test microalgae**.**

**Table 1:** HPLC analysis for phytohormones content of *Chlorella vulgaris*, *Nanochloropsis salina*, and *Arthrospira platensis*. Data represent mean of 3 replicatrs ± SD

Growth regulators	*Chlorella vulgaris*	*Nannochloropsis salina*	*Arthrospira platensis*
GA_3_ (mg100 g^−1^ d.wt.)	52.10 ± 1.21	74.85 ± 2.31	49.44 ± 0.83
IAA (µg 100 g^−1^ d.wt.)	26.76 ± 1.14	34.57 ± 1.09	26.29 ± 1.04
ABA (µg 100 g^−1^ d.wt.)	* N.D	* N.D	* N.D

*IAA* indole-3-acetic acid, *GA*_*3*_ gibberellic acid, *ABA* Abscisic acid

^*^Not detected

### Growth parameters

**Figure 1:**
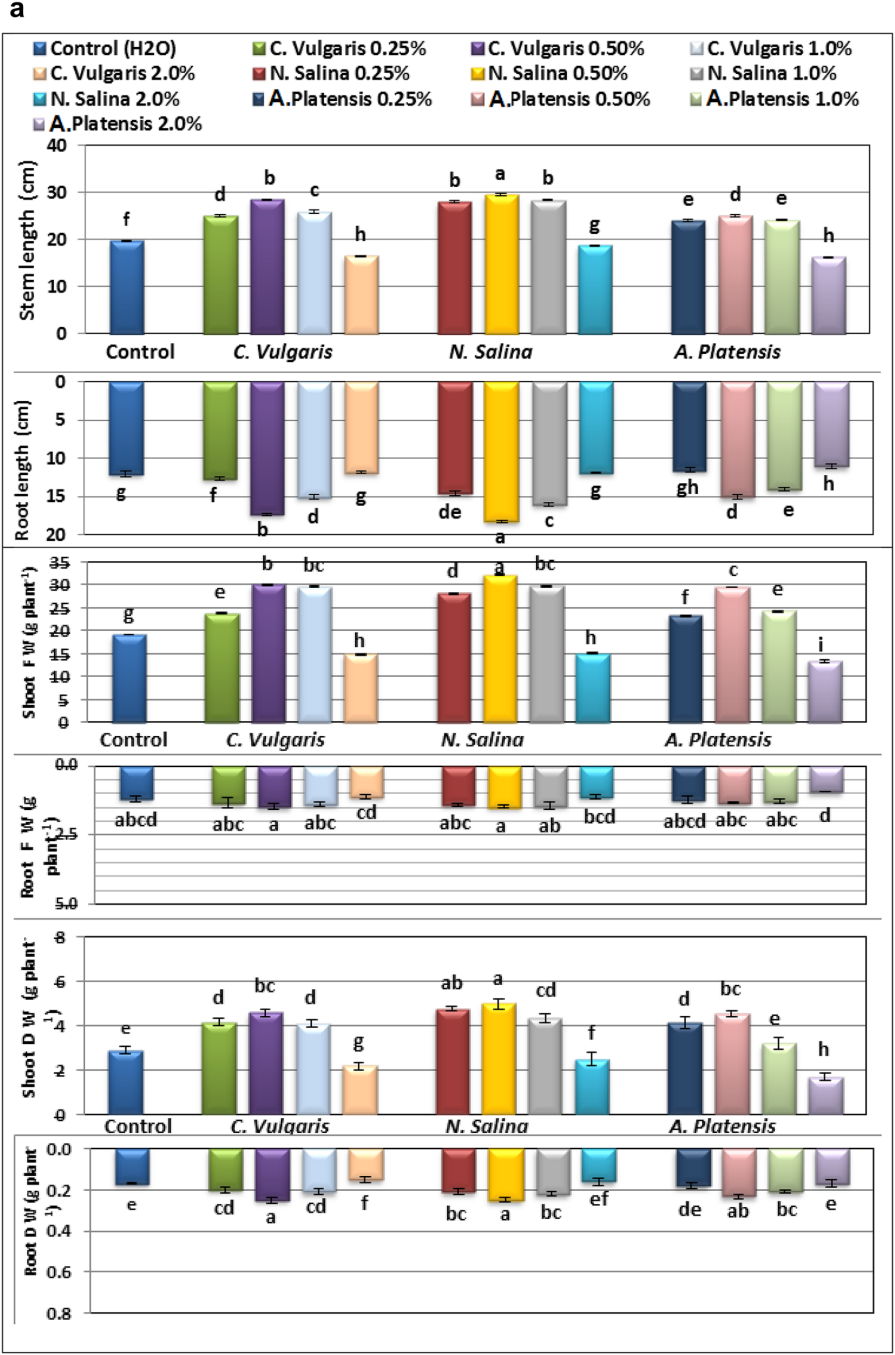

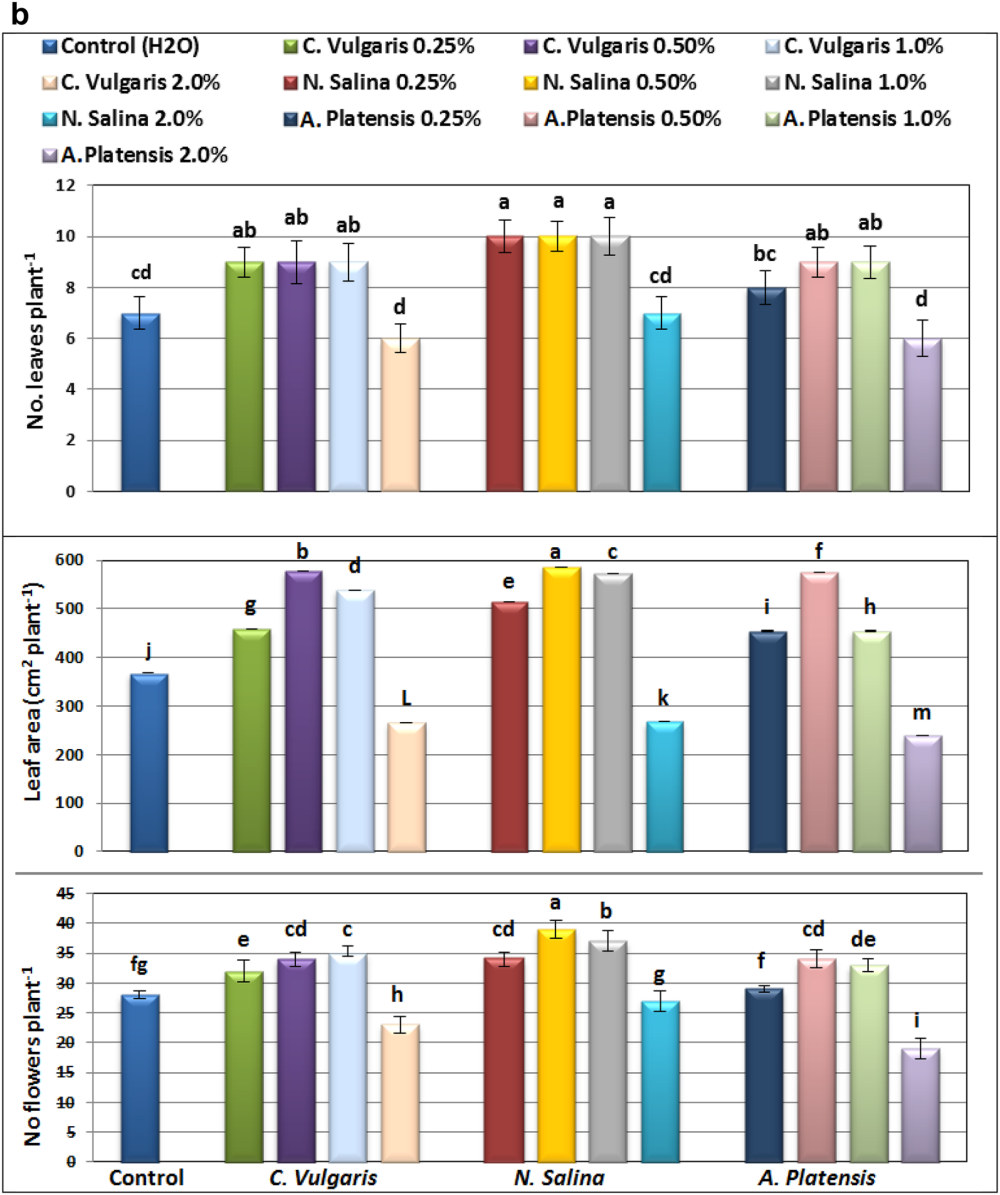
Effect of foliar spray with *Chlorella vulgaris*, *Nannochloropsis salina*, and *Arthrospira platensis* extracts at 0.0, 0.25, 0.5, 1.0, and 2.0% on growth criteria of common bean (var. Bronco) at 40 days after sowing. Different letters indicate significant differences between treatments (Duncan test *p* ≤ 0.05). Vertical bars represent SE.

**Figure 2:**
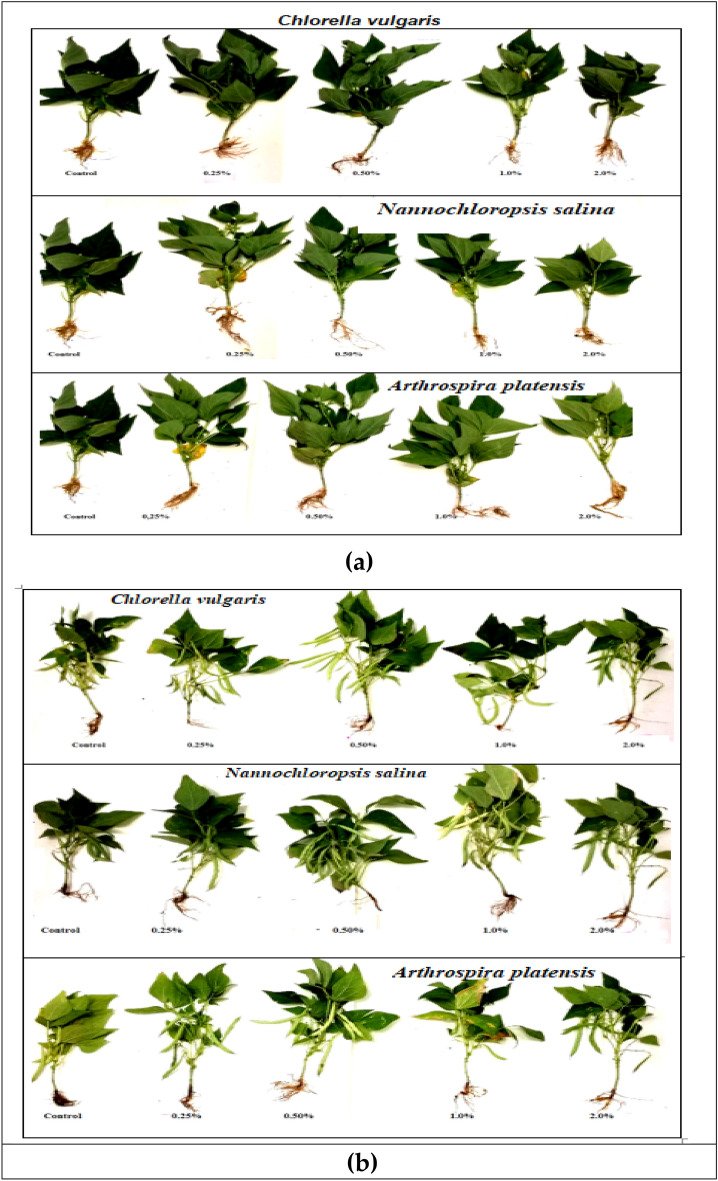

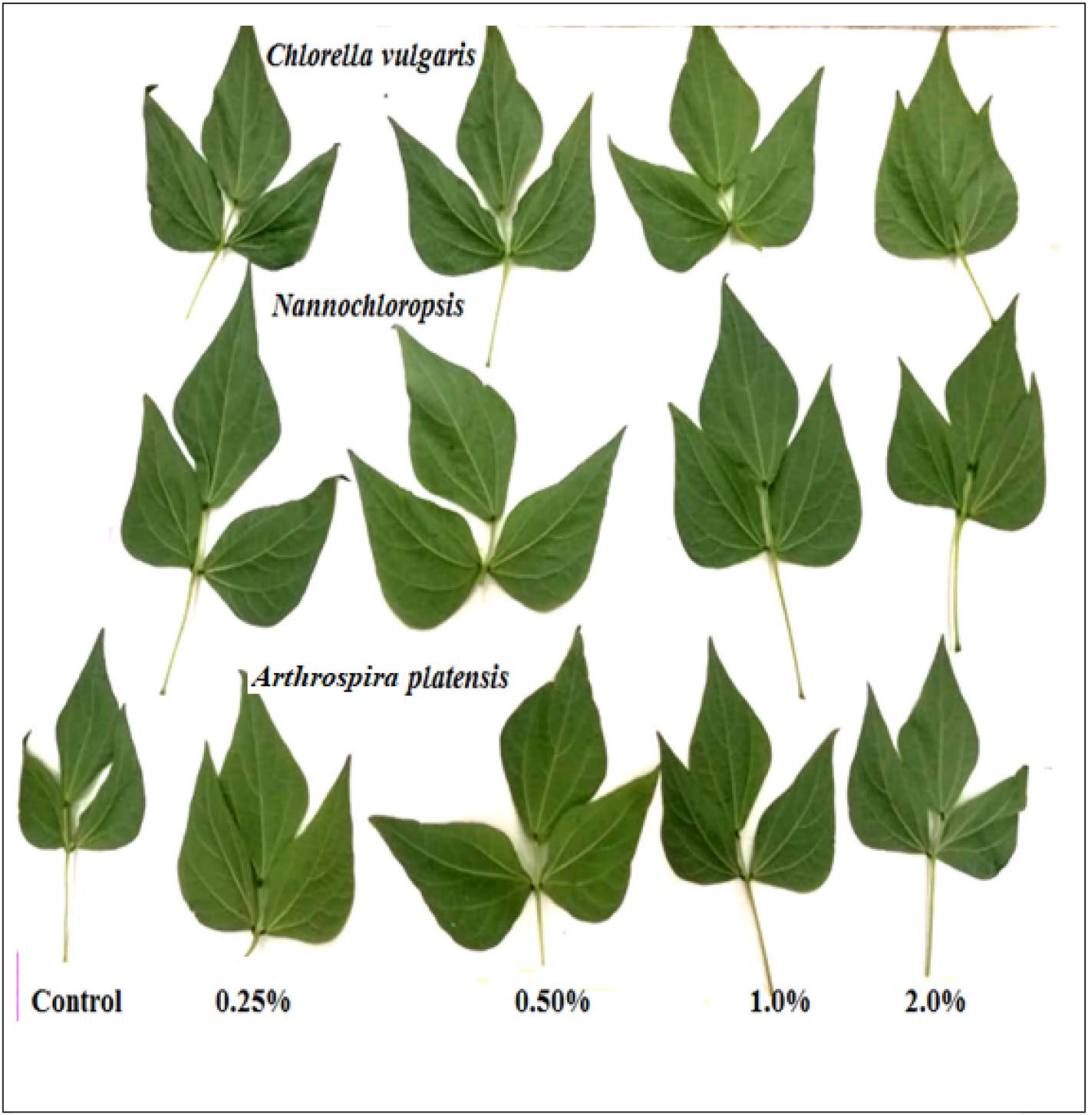
Effect of foliar spray with *Chlorella vulgaris*, *Nannochloropsis salina*, and *Arthrospira platensis* extracts at 0.0, 0.25, 0.5, 1.0, and 2.0% on growth criteria of common bean (var. Bronco) at 40 days after sowing. Different letters indicate significant differences between treatments (Duncan test *p* ≤ 0.05). Vertical bars represent SE.

**Figure 3:**
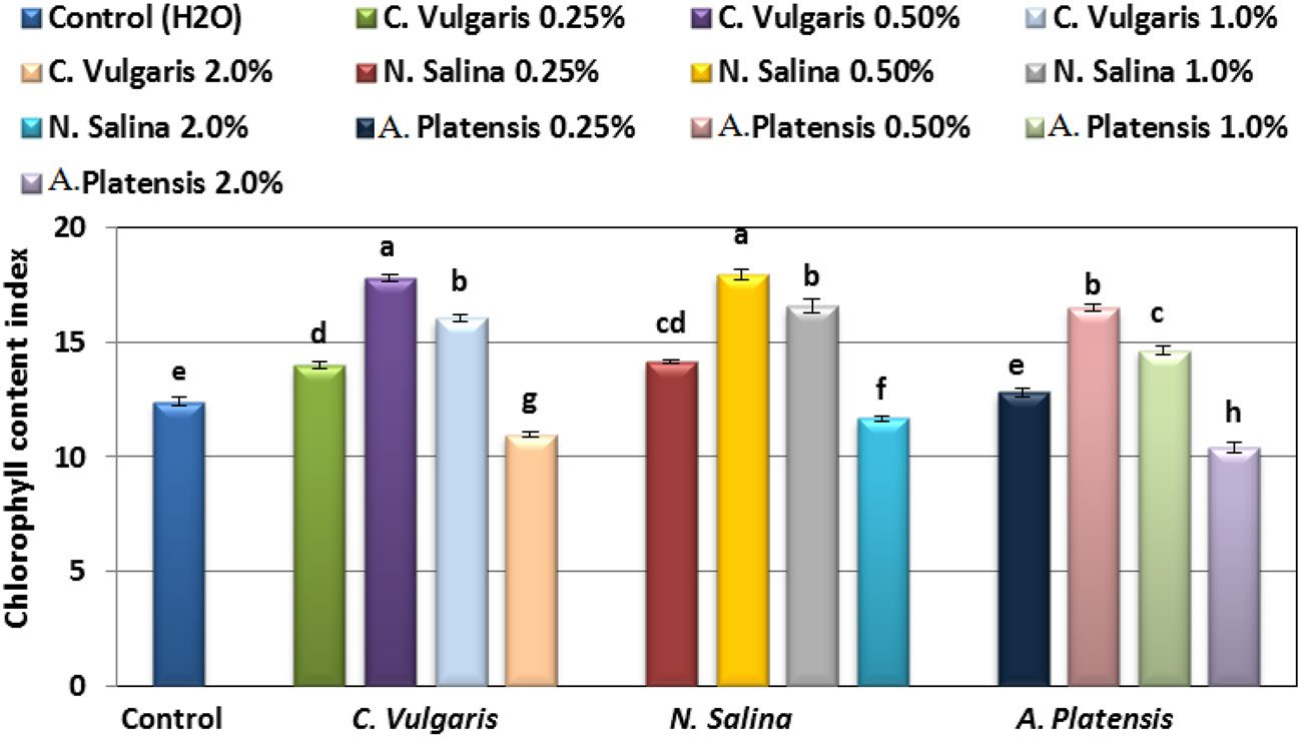
Effect of foliar spray with *Chlorella vulgaris*, *Nannochloropsis salina*, and *Arthrospira platensis* extracts, each at (0.0, 0.25, 0.50, 1.0, and 2.0%) on growth (**a**) and yield (**b**) of common bean (var. Bronco) at 40 and100 days after sowing, respectively.

**Figure 4:**
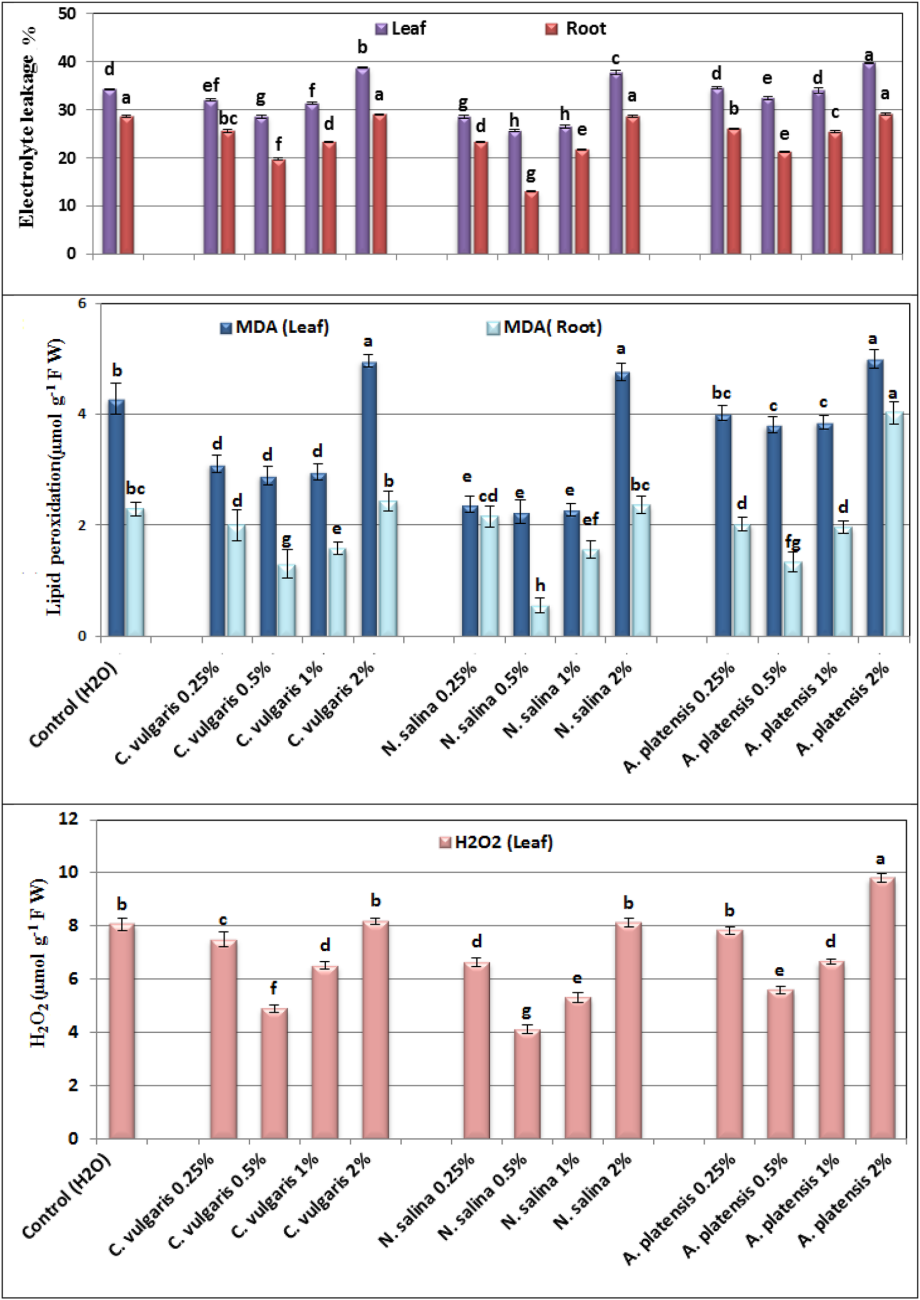
Effect of foliar spray with *Chlorella vulgaris*, *Nannochloropsis salina*, and *Arthrospira platensis* extracts, each at (0.0, 0.25, 0.50, 1.0, and 2.0%) on leaf of common bean (var. Bronco) at 40 DAS.

Results in Figs. 1, 2, 3 and 4 indicate that foliar application of *C. vulgaris*, *A. platensis****,*** and* N. salina* extracts up to 1.0% significantly promoted almost all growth criteria including plant height, root length, number of leaves and flowers per plant, both fresh and dry weights of shoot and root (g plant^−1^), compared to the corresponding untreated common bean plants.

*Nannochloropsis* demonstrated superior effectiveness compared to *Chlorella* and *Arthrospira* in enhancing the vegetative growth of common bean plants. The most significant improvement in growth traits such as PH, RL, LN, LA, FW, and DW was observed with a concentration of 0.5% *Nannochloropsis*. Following closely behind, *Chlorella* at a concentration of 0.5% also resulted in significant growth improvement for all parameters measured (Figs. 1, 2, 3 and 4). Specifically, *Nannochloropsis* at a concentration of 0.5% exhibited the most notable enhancement in root (47.06%) and shoot (73.26%) dry weight per plant compared to control plants. This suggests that *Nannochloropsis* has the highest potential for promoting growth in common bean plants.

Conversely, foliar application of *S. platensis****,**** C. vulgaris,* and *N. salina* extracts at a concentration of 2.0% has a detrimental effect on the growth of common bean plants.* S. platensis *at 2.0% significantly decreased growth criteria, especially, shoot dry weight plant^-1^ decreased by -40.97% compared to untreated group.

### Chlorophyll content index

The chlorophyll content index (CCI) increased significantly by application of *Chlorella, Nannochloropsi*s *and Arthrospira* extracts at 0.25–1.0% concentrations, and decreased at 2.0% level, in comparison with control group.

The maximum values of CCI (17.95. and 17.81%) was obtained at 0.50% *N. salina*, followed by *C.vulgaris* extract, but the lowest CCI (10.43, 10.96, and 11.69%) was recorded at 2.0% *Arthrospira, Chlorella,* and *Nannochloropsi*s extracts, respectively.

**Figure 5:**
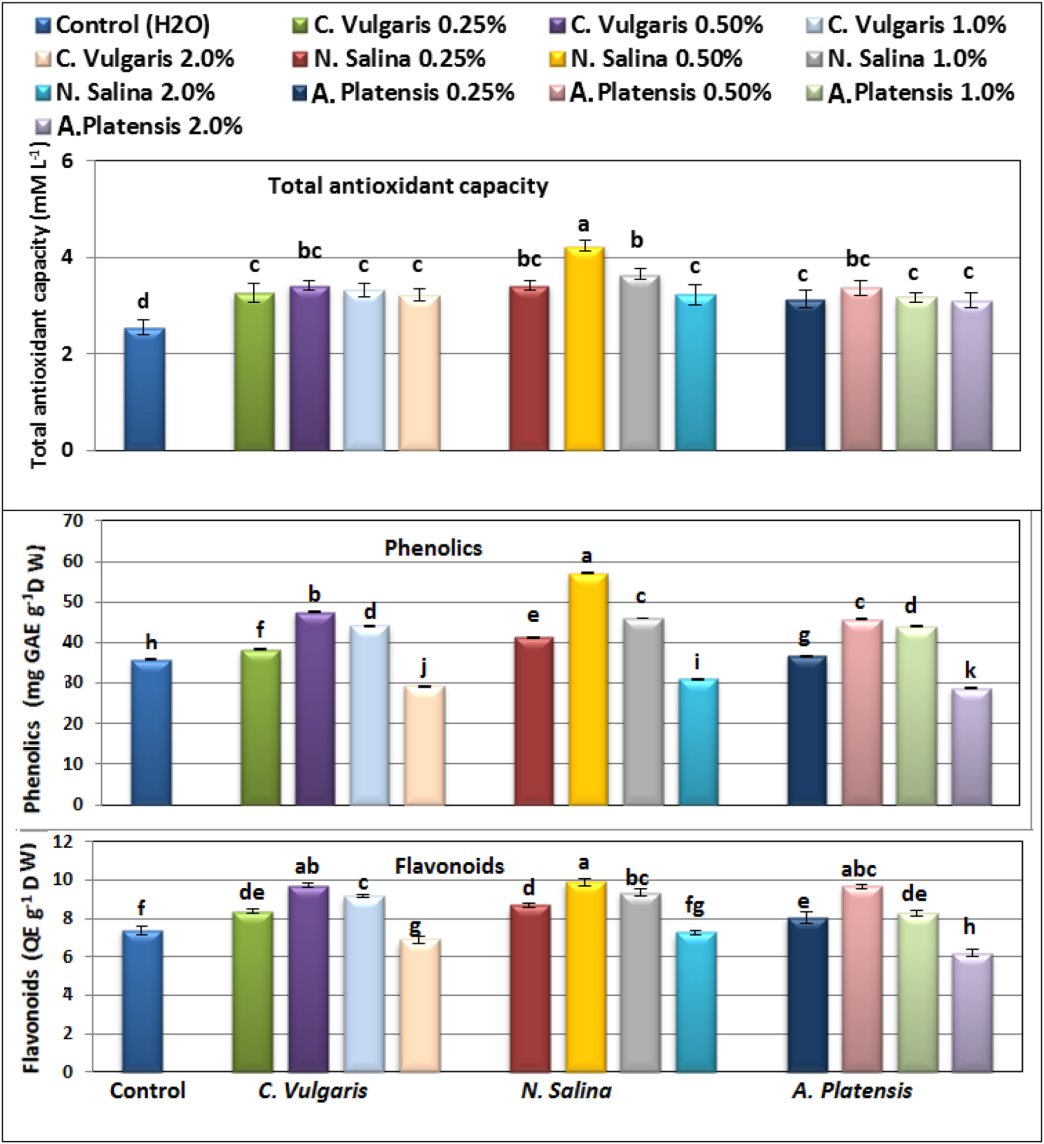
Effect of foliar spray with *Chlorella vulgaris*, *Nannochloropsis salina*, and *Arthrospira platensis* extracts at 0.0, 0.25, 0.50, 1.0, and 2.0% on chlorophyll content index in the leaves of common bean (var. Bronco) plants at 40 days after sowing. Each result is a mean of 10 replicates. Statistical analysis was carried out using Duncan test. Vertical bars represent SE.

Generally, CCI values of common bean from *N. salina* treatments was greater than *C. vulgaris* and *Arthrospira* extracts at all used concentrations (Fig. 5)*.*

### Oxidative stress markers

The data show that the use of *Chlorella*,* Nannochloropsis,* and *Arthrospira* extracts in concentrations ranging from 0.25% to 1.0% had a significant impact on reducing malondialdhyde (MDA) level, a byproduct of lipid peroxidation caused by free radicals. Additionally, these extracts decreased the percentage of electrolyte leakage (EL), which is an indicator of permeability of cell membrane in both the fresh leaves and roots. Furthermore, the extracts reduced the hydrogen peroxide (H_2_O_2_) content, which is a highly damaging form of reactive oxygen species, in the leaves of common bean plants, relative to control group (Fig. 4).

Within the different applied treatments, the percentage of EL and MDA content were higher in the leaves than root of common bean plants. The minimum values of MDA (2.24, and 0.56 µmol g^−1^ FW. equiv), relative permeability (25.71, and 13.04%), in leaves and root, and H_2_O_2_ levels (4.11 µmol g^−1^ f.wt. equiv.) in leaves were recorded at 0.5% *N. salina* compared with (4.28, and 2.30 µmol g^−1^ FW. equiv., 34.25, and 28.71%, and 8.07 µmol g^−1^ FW. equiv.) for their respective controls.

**Figure 6:**
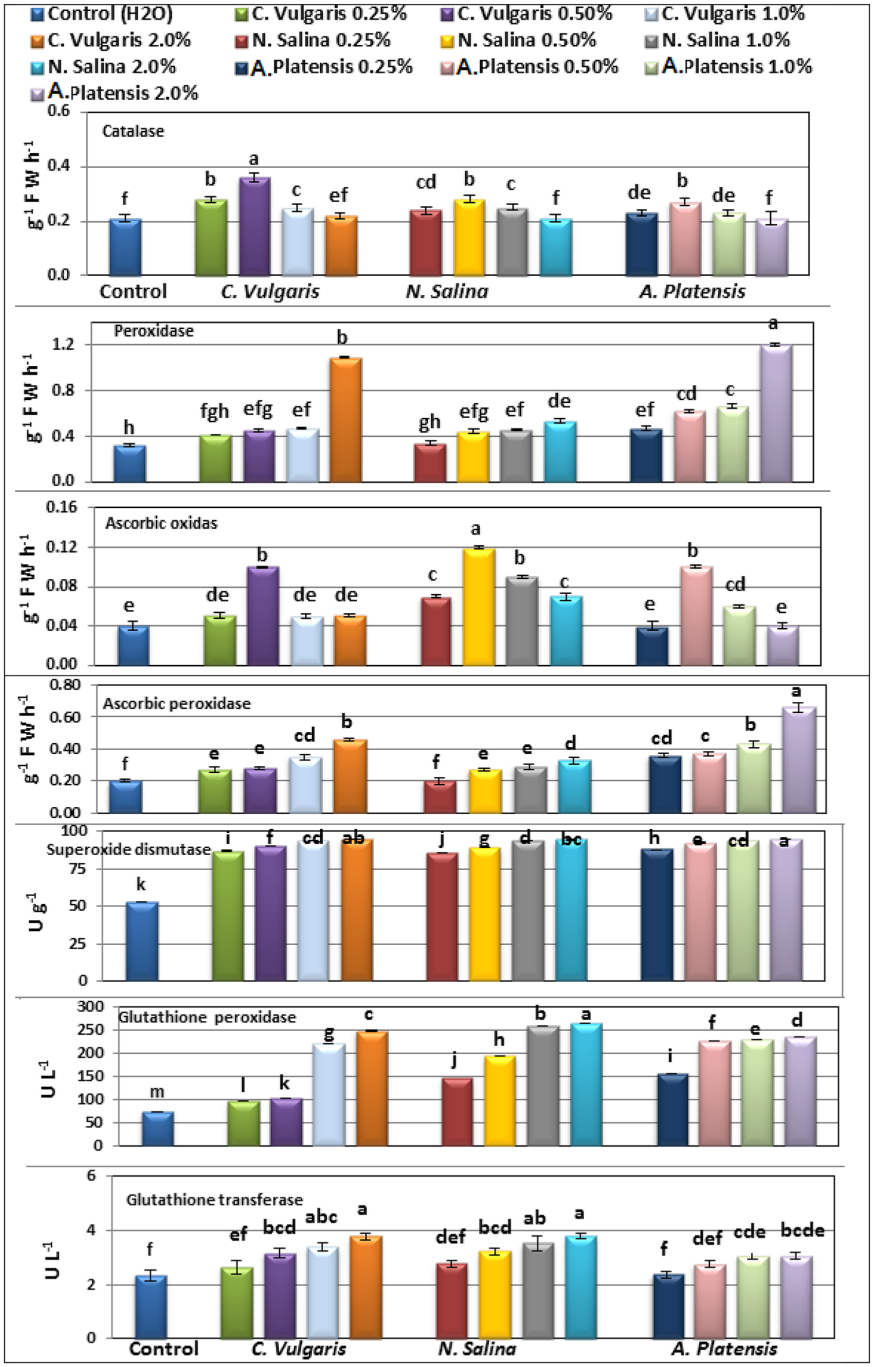
Effect of foliar spray with *Chlorella vulgaris*, *Nannochloropsis salina*, and *Arthrospira platensis* extracts at 0.0, 0.25, 0.50, 1.0, and 2.0% on the electrical conductivity percentage, lipid peroxidation (µmol g^−1^ F W) in the fresh leaves and roots, and the content of hydrogen peroxide (H_2_O_2_) (µmol g^−1^ F W) in leaf tissue of common bean (var. Bronco) plants at 40 days after sowing. Each result is a mean of 3 replicates. Vertical bars represent SE.

However, the peroxidative damage, relative permeability in leaf, and root tissues, and H_2_O_2,_ content in leaf reached their highest levels when three microalgal extracts, notably *Arthrospira* at a concentration of 2%, were administered. These levels recorded (5.00, and 4.02 µmol g^−1^ FW. equiv.) for peroxidative damage, (39.73, and 29.17%) for relative permeability, and (9.80 µmol g^−1^ FW. equiv.) for H_2_O_2_ content in leaf as depicted in Fig. 6.

### Non enzymatic antioxidants

According to the findings, foliar spray with *Chlorella*,* Nannochloropsis,* and *Arthrospira* extracts at 0.25 – 1.0% concentrations can significantly enhance the level of non-enzymatic antioxidants including total antioxidant capacity, flavonoids, and total phenols, relative to untreated common bean plants (Fig. 6),

Among the three extracts, *N. salina* at a concentration of 0.5% showed the most promising rise in antioxidant capacity (4.25 mM L^−1^), phenolic (57.01 mg GAE g^−1^), and flavonoid (9.90 mg QE g^−1^) compared to (2.55 mM L^−1^, 35.77 mg GAE g^−1^, and 7.39 mg QE g^−1^) for their respective untreated bean plants. *C. vulgaris* at 0.5%, then *S. platensis *extracts, also exhibited increases in these antioxidant components, but to a lesser extent.

**Figure 7:**
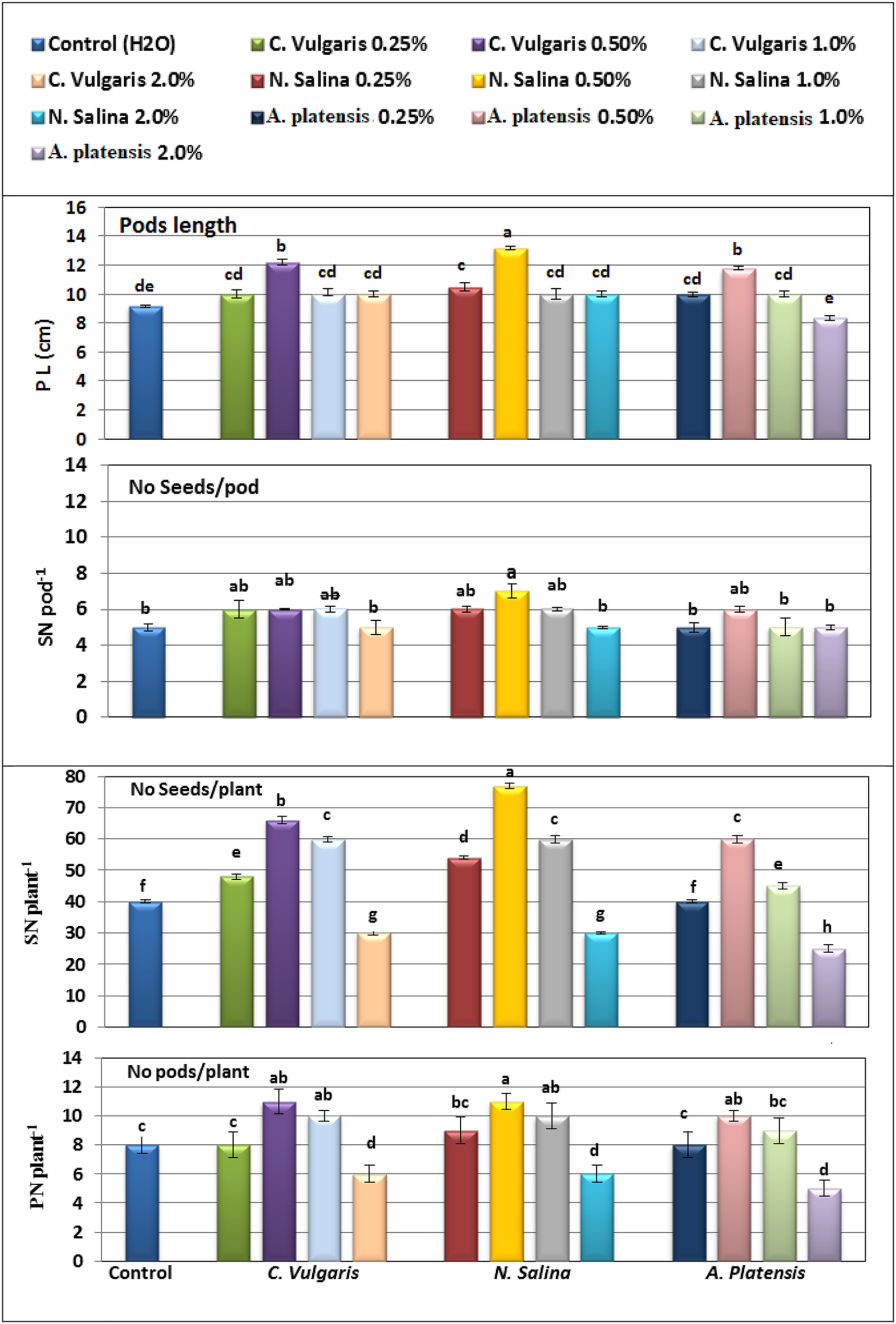

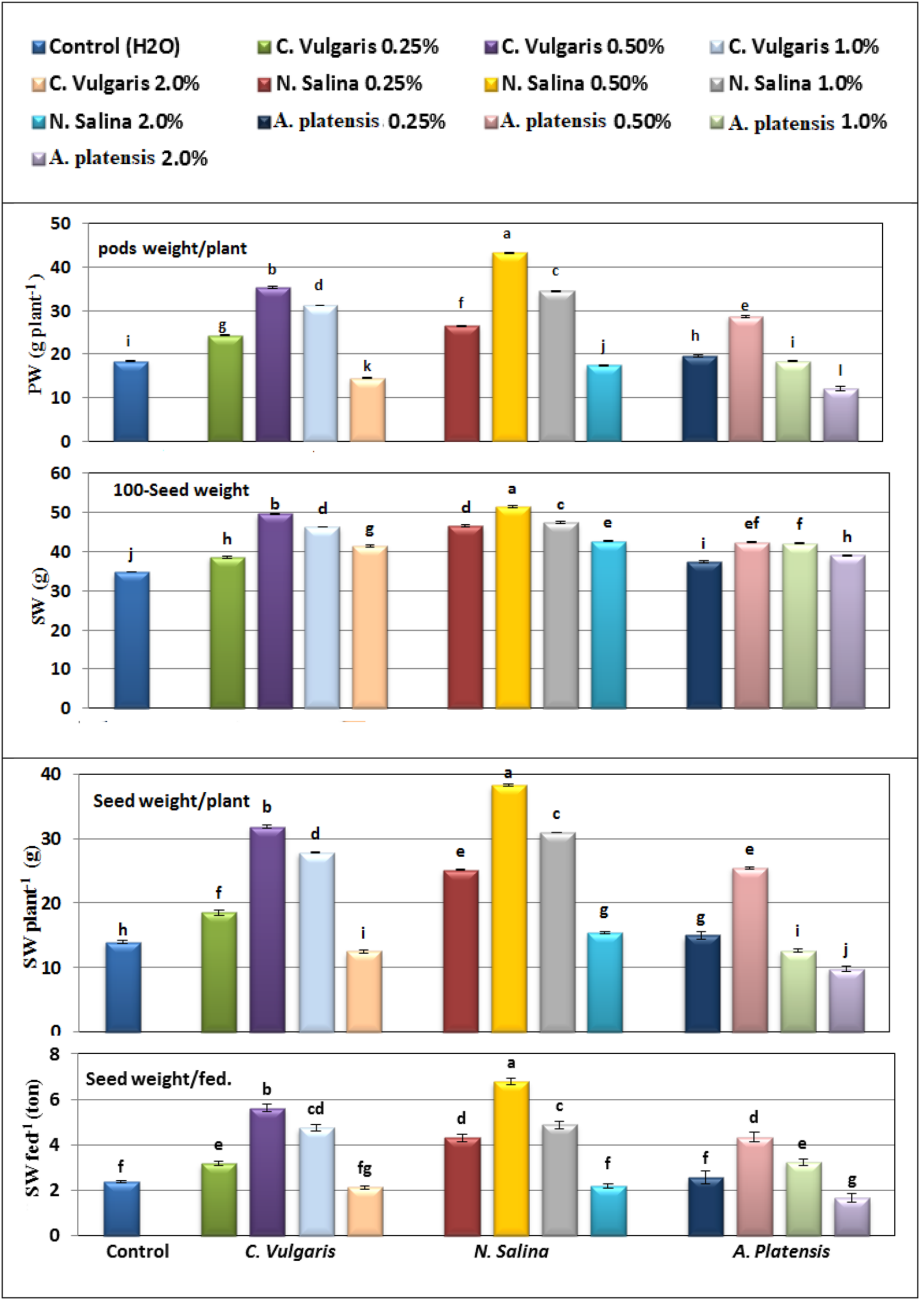
Effect of foliar spray with *Chlorella vulgaris*, *Nannochloropsis salina*, and *Arthrospira platensis* extracts at 0.0, 0.25, 0.5, 1.0, and 2.0% on seed yield characters of common bean (var. Bronco) at 100 days after sowing. Different letters indicate significant differences between treatments (Duncan test *p* ≤ 0.05). Vertical bars represent SE.

However, higher concentrations of three algal extracts at 2.0% lead to a decrease in the levels of these measured non-enzymatic antioxidants in the leaves of common bean plants. The minimum values of total antioxidant capacity (3.11 mM L^−1^), total phenols (28.75 mg GAE g^-1^), and flavonoids (6.20 mg QE g^−1^) was observed by application of *S. platensis* at 2%, followed by *C. vulgaris*, then *N. salina* extracts at the same concentration, relative to their corresponding controls (Fig. 7).

### Antioxidant enzymes

**Figure 8:**
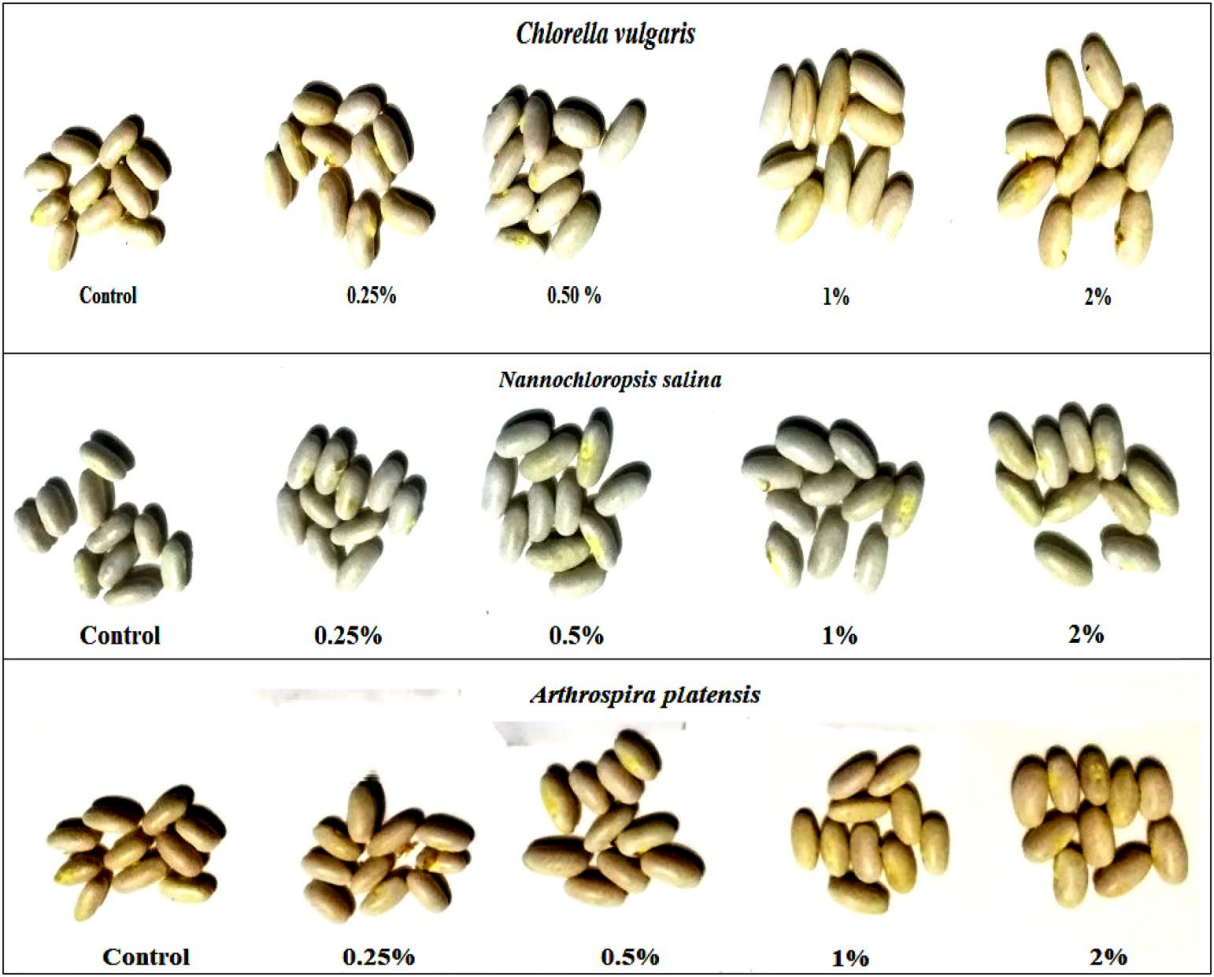
Changes in the activities of the antioxidant enzymes catalase, peroxidase and ascorbic oxidase, ascorbic peroxidase (g FW. equiv/ h), superoxide dismutase (U/g), glutathione peroxidase (U/L) and glutathione transferase (U/L) of fresh leaf tissue of common bean (var. Bronco) plants at 40 DAS as affected by foliar spray with *C.vulgaris*, *N. salina,* and *A*. *platensis* extracts at 0.0, 0.25, 0.5, 1.0, and 2.0%. The results are expressed as means of three replicates. Different letters show significant variation at 0.05 P. Vertical bars represent SE.

The results depicted in Figure 8 demonstrate a linear improvement in the activity of various antioxidant, including peroxidase (POX), ascorbic peroxidase (APX), superoxide dismutase (SOD), glutathione peroxidase (GSH-Px), and glutathione transferase (GSH-T), following the application of *C. vulgaris*, *S. platensis*, and* N. salina* extracts at concentrations ranging from 0.25 to 2.0%. Conversely, the activity of catalase (CAT) and ascorbic oxidase (AOX) initially increased up to a 1% concentration of the three algal extracts, but subsequently decreased in common bean compared to untreated group.

Moreover, the foliar spray of *Chlorella*,* Nannochloropsis,* and *Arthrospira* extracts at concentrations of 0.25 –2.0% led to a rise in the activity of CAT, POX, AOX, APX, SOD, GSH-Px, and GSH-T in common bean leaves relative to the control group.. This stimulating effect was most prominent for POX, APX, SOD, GSH- Px, and GSH-T antioxidant at a 2.0% concentration of the three algal extracts, while, the highest activity of CAT and AOX was observed at a 0.5% concentration of *Chlorella*, and *Nannochloropsis*, respectively (Figure 8).

Furthermore, the effectiveness of *Arthrospira* extract at 2.0% concentration was found to be superior to that of *Chlorella* and *Nannochloropsis* at the same concentration in enhancing the activity of POX, APX, and SOD in common bean plants. Additionally, the highest activities of GSH-T, and GSH-Px were observed when* Nannochloropsis* was applied at a concentration of 2.0% compared to the control groups (Fig. 8).

### Yield and Yield Component

The data presented in Fig. 3 and 7 demonstrate that the utilization of extracts from *C. vulgaris*, *A. platensis*, and* N. salina* at 0.25 to 1.0% levels, resulted in an increase in common bean yield. This increase was observed in various plant characteristics such as pod length (PL), number of pods per plant (PN P^−1^), seeds per pod (SN P^−1^) and seeds per plant (SN Pl^−1^), pod weight per plant (PW P^−1^), seed index (SI) (100-SW), seed weight per plant (SY P^−1^), and seed yield per feddan (SY F^−1^) at the harvest date. However, at a higher concentration of 2.0%, the application of these extracts led to a reduction in yield. The most promising outcome was observed in SY F^−1^ with the application of 0.5% *N. salina* extract.

In the study, it was found that using *Chlorella* and *Arthrospira* extracts at concentrations up to 1.0% increased common bean yield compared to untreated controls. Specifically, the yield parameters of PN P^−1^, PL, SN P^−1^, PW P^−1^, SI, SY P^−1^, and SY F^−1^ were all significantly higher with the application of these extracts.

Moreover, when *Chlorella* extract was used at 0.5%, there was a remarkable increase in SY F^−1^, which was 135.75% higher than the control. However, it was observed that *Nannochloropsi*s extract had an even greater impact on common bean yield. The application of *Nannochloropsi*s at 0.5% resulted in the highest increase in yield parameters, with SI, SY P^−1^, and SY F^−1^ increasing by 47.99%, 184.88%, and 185.01% respectively, relative to their respective controls (Fig. 7).

On the other hand, the lowest yield attributes, specifically, SY P^−1^ decreased by 29.78% and SY F^−1^ decreased by 29.75% compared to controls were observed when 2.0% *Arthrospira* extract was used, followed by *Chlorella* and* Nannochloropsi*s extracts at the same concentration (Fig. 7).

### Proximate chemical composition of seed

In the study, the proximate compositions of seeds were analyzed after being spray common bean plants with extracts from *C. vulgaris*,* A. platensis*, and *N. salina* at concentrations ranging from 0.25 to 2.0%. Results from the harvest date demonstrated significant variations in the moisture content, total ash, total fat, crude fiber, crude protein, total carbohydrate, and energy of the seeds among the different treatments, as shown in Table 2 and Fig. 10.

**Table 2:** Effect of foliar spray with *Chlorella vulgaris*, *Nannochloropsis salina*, and *Arthrospira platensis* extracts at 0.0, 0.25, 0.5, 1.0, and 2.0% on proximate chemical composition percentage (moisture content, total ash, total fat, crude fiber, crude protein, total carbohydrate, and energy (Kcal g^−1^)) of yielded seeds of common bean (var. Bornco) at 100 DAS, each result is a mean of three replicates ± SD

Treatments %	Energy	Moisture	Total ash	Total fat	Crude fiber	Crude protein	Total Carbohydrate
	(Kcal g^−1^)	(%)					
Control (H_2_O)	353.01^i^ ± 0.056	11.66^bcd^ ± 0.190	1.00^fg^ ± 0.029	0.73^ghi^ ± 0.098	5.23^ J^ ± 0.060	26.20^j^ ± 0.115	60.41^d^ ± 0.043
*C. vulgaris* 0.25%	370.35^a^ ± 0.023	11.58^ cd^ ± 0.164	1.22^de^ ± 0.046	4.31^a^ ± 0.063	11.05^a^ ± 0.057	31.94^ h^ ± 0.023	50.95^i^ ± 0.028
*C. vulgaris* 0.5%	362.75^b^ ± 0.092	10.98^f^ ± 0.078	1.77^a^ ± 0.103	2.75^b^ ± 0.144	8.19^d^ ± 0.109	36.72^b^ ± 0.103	47.78^k^ ± 0.051
*C. vulgaris* 1.0%	356.43^e^ ± 0.026	11.24^def^ ± 0.138	1.34^cd^ ± 0.080	1.35^e^ ± 0.046	6.80^f^ ± 0.057	34.83^e^ ± 0.040	51.24^h^ ± 0.034
*C. vulgaris* 2.0%	351.63^j^ ± 0.040	12.04^ab^ ± 0.031	0.89^g^ ± 0.028	0.67^ghi^ ± 0.036	5.08^j^ ± 0.052	24.14^ l^ ± 0.034	62.26^b^ ± 0.075
*N. salina* 0.25%	361.91^c^ ± 0.023	11.24^def^ ± 0.046	1.07^ef^ ± 0.040	2.23^C^ ± 0.040	9.54^b^ ± 0.109	32.73^ g^ ± 0.098	52.73^g^ ± 0.098
*N. salina* 0.5%	361.52^d^ ± 0.032	10.33^ g^ ± 0.155	1.79^a^ ± 0.077	2.00^d^ ± 0.044	7.32^e^ ± 0.063	38.31^a^ ± 0.121	47.57^k^ ± 0.056
*N. salina* 1.0%	354.54^ g^ ± 0.037	11.09^ef^ ± 0.115	1.50^b^ ± 0.043	0.98^f^ ± 0.023	6.12^ h^ ± 0.054	35.08^d^ ± 0.060	51.35^h^ ± 0.046
*N. salina* 2.0%	351.52^j^ ± 0.017	11.97^abc^ ± 0.121	0.95^fg^ ± 0.017	0.64^hi^ ± 0.034	4.63^ k^ ± 0.075	25.22^ k^ ± 0.040	61.22^c^ ± 0.040
*A. platensis* 0.25%	355.96^f^ ± 0.037	11.57^ cd^ ± 0.109	1.04^dfg^ ± 0.029	1.28^e^ ± 0.063	8.86^c^ ± 0.072	28.01^i^ ± 0.104	58.10^e^ ± 0.057
*A. platensis* 0.5%	353.40^ h^ ± 0.173	11.29^def^ ± 0.173	1.41^bc^ ± 0.049	0.84^ fg^ ± 0.034	6.58^ g^ ± 0.063	35.92^c^ ± 0.046	50.54^j^ ± 0.052
*A. platensis* 1.0%	353.21^h^ ± 0.040	11.45^de^ ± 0.089	1.26^cd^ ± 0.040	0.81^fgh^ ± 0.020	5.83^i^ ± 0.040	33.17^f^ ± 0.075	53.31^f^ ± 0.029
*A. platensis* 2.0%	351.04^k^ ± 0.049	12.25^a^ ± 0.173	0.69^h^ ± 0.023	0.56^i^ ± 0.017	4.62^ k^ ± 0.046	23.88^ m^ ± 0.063	62.62^a^ ± 0.032
L.S.D at 5%	0.19	0.33	0.19	0.20	0.22	0.25	2.60

Maximum and minimum values are underlined

Statistical analysis was carried out using Duncan test

Different letters show significant variation at 0.05 P

The foliar application of *Chlorella*, *Nannochloropsis*, and *Arthrospira* extracts at concentrations up to 1.0% significantly increased the levels of crude protein (CP), total fat (TF), crude fiber (CF), ash, and energy in the seeds, while simultaneously decreasing the moisture content and total carbohydrates (TC) compared to the control group.

Among the different treatments, it was observed that the most effective treatment in increasing the percentage of crude protein and ash, while minimizing moisture content, was the use of 0.5% *N. salina*. Following this, the application of *C. vulgaris* at the same concentration showed similar effects, and lastly, *A. platensis* at the same concentration exhibited a slightly lower impact on these parameters.

*Nannochloropsis* at 0.5% had the highest values of crude proteins (38.31%), and ash (1.79%), as well as high fat (2.00%), crude fibers (7.32%), and energy (361.52 kcal) with the lowest total carbohydrates (47.57%), and lowest moisture content (10.33%)**.** On the contrary, *S. platensis* at 2.0% had the lowest level of CP (23.88%), TF (0.56%), leading to lowest energy (351.04 kcal), Also, lowest CF (4.62%), and ash (0.69%), but the highest value of TC (62.62%) in common bean seeds. In addition, the results demonstrated that *C. vulgaris* at 0.25% tended to exhibit the highest TF (4.31%)**,** and high protein (31.94%) contents, leading to the highest energy (370.35 kcal) and also exhibited the highest dietary fiber (11.05%)**.**

**Figure 9:**
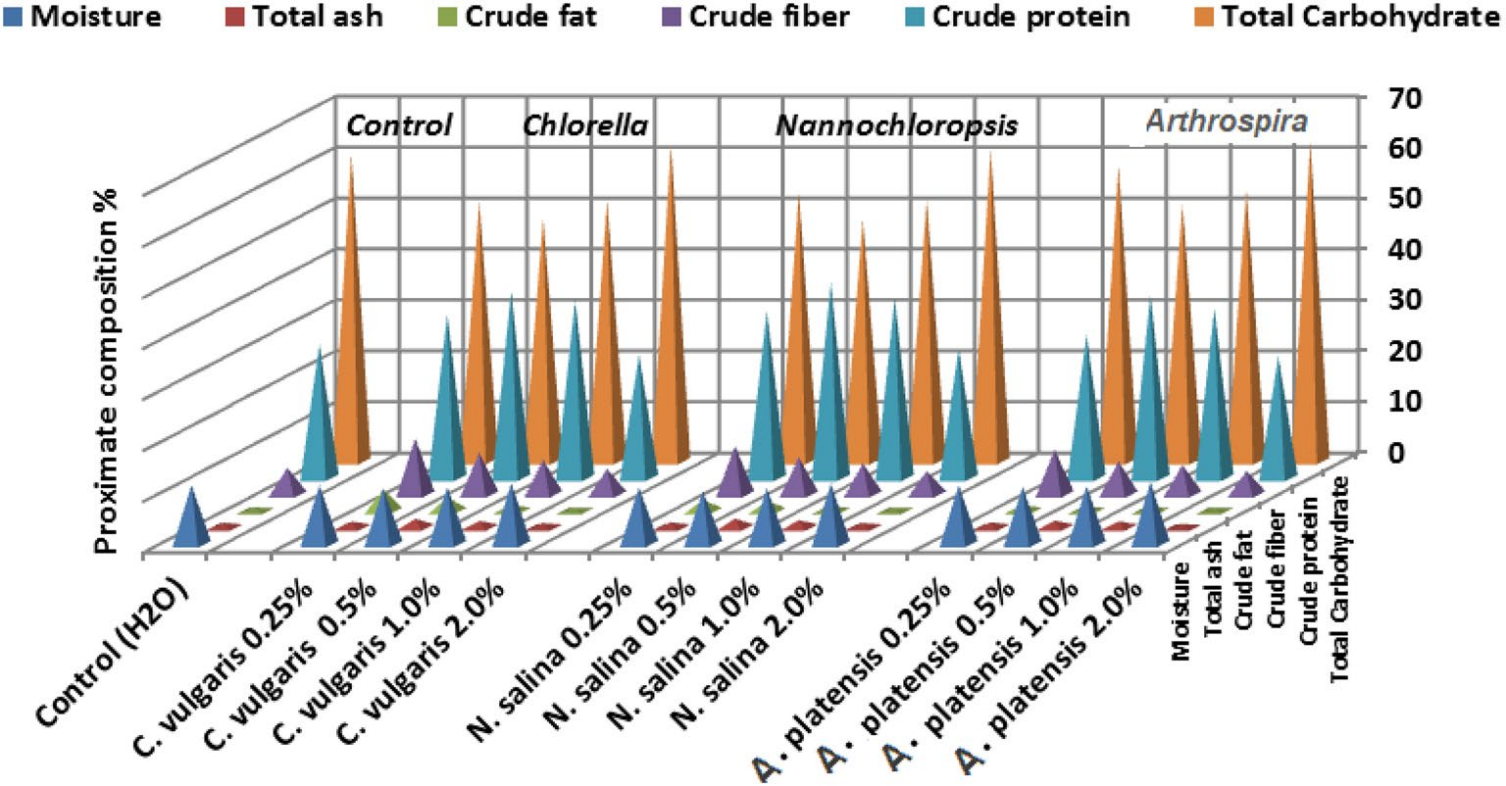
Proximate chemical composition (moisture, total ash, total fat, crude fiber, total protein, and total carbohydrate) percentage in yielded seeds of common bean (var. Bornco) plants foliar sprayed with *Chlorella vulgaris*, *Nannochloropsis salina*, and *Arthrospira platensis* extracts at 0.0, 0.25, 0.5, 1.0, and 2.0% at 100 days after sowing, each result is a mean of three replicates.

On the average, *N. salina* treatments improved the nutritive values of common bean seeds than *C. vulgaris* and *S. platensis* ones. The seeds showed significant differences in in all investigated variables (i.e., MC, CP, CF, TF, ash, TC, and E) between *C. vulgaris*, *N. salina* and *A. platensis* treatments (Table 2 and Fig. 9).

### Mineral content of seed

**Figure 10:**
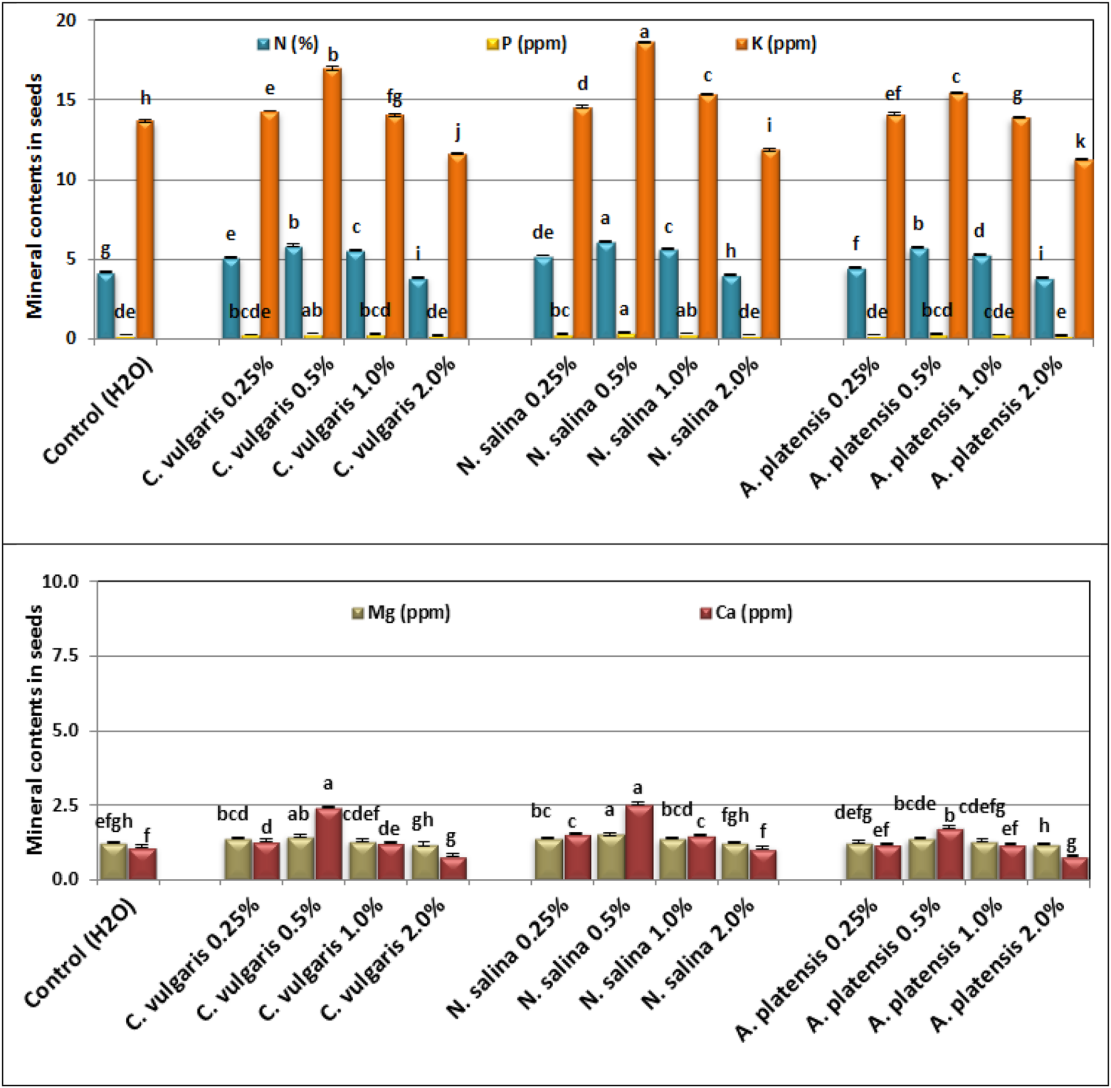
Effect of foliar spray with *Chlorella vulgaris*, *Nannochloropsis salina*, and *Arthrospira platensis* extracts at 0.0, 0.25, 0.5, 1.0, and 2.0% on seed mineral (nitrogen%, phosphorus, potassium, and magnesium in ppm) concentrations of common bean (var. Bronco) plants at 100 days after sowing.

Foliar application of *C. vulgaris*, *S. platensis*, and *N. salina* up to 1% concentrations significantly increased N%, P, K, Ca, and Mg contents (ppm) in common bean seeds at 100 DAS. On the other hand, a slight decrease was observed in N%, P, K, Ca and Mg level at 2% algal extract. In general, *Nannochloropsis* was more superior than *Chlorella*, and *Arthrospira* at 0.5–2.0% in raising the mineral content of seeds. *Nannochloropsis* at 0.5% showed maximal contents of N, P, K, Ca, and Mg being 6.12%, 0.37, 18.61, 1.54 and 2.54 ppm, respectively in treated plants, followed by *Chlorella* at 0.5%. Whereas, plants sprayed with *Arthrospira* at 2% recorded minimum values of N%, P, K, Ca, and Mg being 3.82%, 0.20, 11.29, 1.19, and 0.82 ppm compared with 4.19%, 0.23, 13.69, 1.27, and 1.12 ppm for their respective controls (Fig. 10).

### Principle component analysis

**Figure 11:**
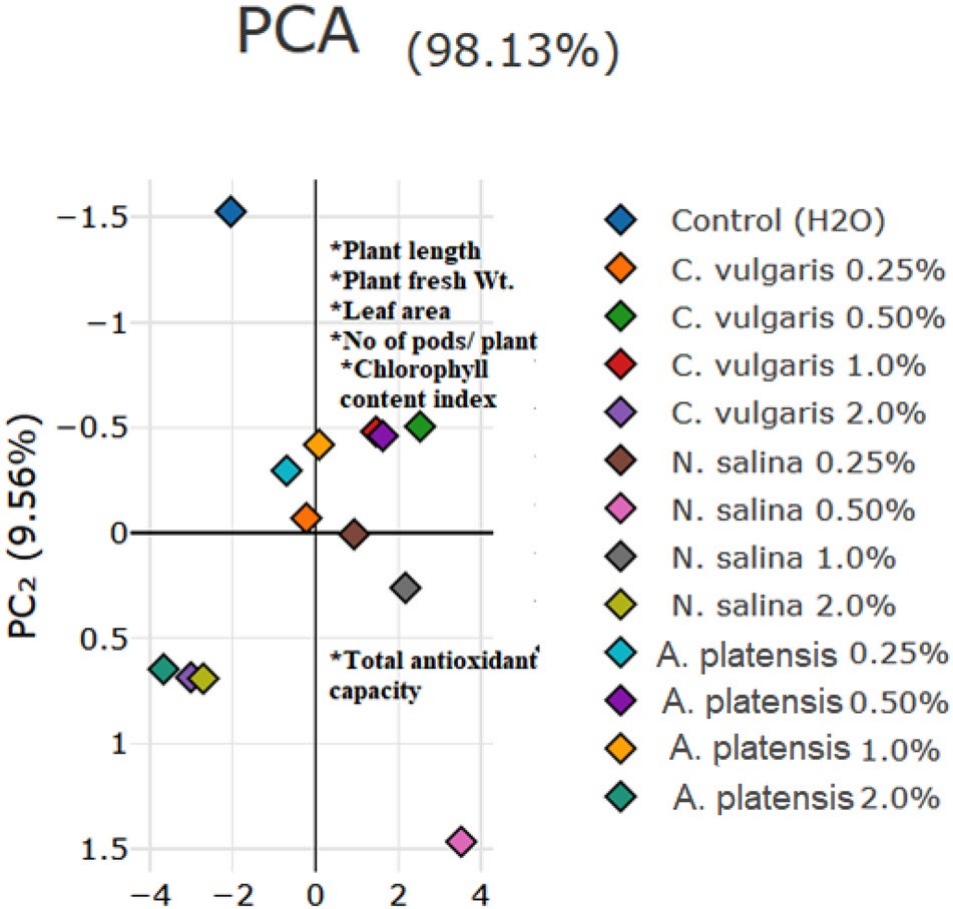
Principle component analysis including plant length, fresh weight. , leaf area, chlorophyll content index and pods number per plant.

The Fig. 11 shows the correlations between each PC and the original variables. Principle parameters included plant length, fresh wt. , leaf area, chlorophyll content index and pods number per plant. Most of the variable were showed significant change sufficient to predict the enhancement of algal extracts to kidney bean plant growth.

## Discussion

In this study, the effectiveness of using microalgae bio-stimulants, specifically *C. vulgaris*, *A. platensis*, and *N. salina* through foliar application, was evaluated on the growth, some metabolic activities and yield of Bronco variety of common beans The application of the 3 microalgal extracts especially improved plant showed positive effects on various growth parameters , this can be attributed to the biologically active compounds present in the algal extracts, particularly phytohormones, which have positive influence on both root and shoot development. Previous research has also shown that a larger root system, facilitated by the action of phytohormones such as auxins, leads to increased nutrients and water uptake from soil, ultimately promoting plant growth and vigor^48,49^. Furthermore, cytokinins enrichment in plant roots can promote the expression of genes encoding for root nitrate and sulfur transporters, resulting in greater nutrient uptake by the plant^50^. In addition, microalgae extracts contain macro and micronutrients, vitamins, polysaccharides, polypeptides, and phytohormones, which trigger metabolic responses like photosynthesis, respiration, and ions uptake^51^. In terms of this aspect, the ability of *Arthrospira* spp. to promote growth is not solely attributed to their hormone composition. Additionally, the nutrients present in the extracts are readily absorbed by the leaves through the stomata and cuticle hydrophilic pore^52^. Applying total polysaccharides extracts solution of *S. platensis* increased root weight, number of nodes, and size of pepper, and tomato plants^53^, Mixed* C. vulgaris* with *Arthrospira platensis* improved growth, and yield characters of rice plants^54^.

Enhancement of growth was also due to the higher content of phytohormones gibberellins [GA_3_], and indole acetic acid in algal extracts. Gibberellins regulate stem elongation leaf expansion, early flowering and seed development^21^. Similarly, as observed in a study conducted by^55^, *C. vulgaris* extract improved growth performance in lettuce seedlings due to hormone-like compounds, and synergisms between various substances. The utilization of hydrolysates from *A. platensis* and *Scenedesmus* spp. at a concentration of 10 g L^−1^ demonstrated enhancements in the root dry matter, number of flowers per plant, flower dry, and fresh weight in petunia plants^21^. The inclusion of *Arthrospira* and *Klamath* algae has the potential to enhance the growth and flowering of *Portulaca grandiflora* plants by increasing the quantity of flowers and prolonging the duration of flowering^56^. Also, *N. salina* was more effective than* C. vulgaris*, and *Enterobacter cloacae* in enhancing leaf, stem and root dry weight and phytochemicals accumulation in *M. oleifera* plants^27^.

On the contrary, when three microalgae extracts are applied to the leaves of common bean plants at a high dose of 2.0%, the stimulating effect is nullified, leading to a significant reduction in various growth parameters. This could be attributed to an imbalance in plant hormones and disruption of mineral element homeostasis, in comparison to the control plants that were not treated with the extracts. These findings align with previous studies that demonstrated the negative impact of applying *A. platensis* extract on radish fresh weight at concentrations exceeding 15%^57^, and the inhibitory effect on *Lupinus luteus* growth at a concentration of 1.0%^58^.

Chlorophyll content index values of common bean were higher when treated with *N. salina* compared to extracts of *C. vulgaris,* and* S. platensis*, across all concentrations used. The application of algal extract at low levels may enhance cellular metabolism, delay aging and chlorophyll degradation, and/or increase chlorophyll biosynthesis by improving nitrogen and magnesium intake; both are important structural components of chlorophyll. Consequently, this results in increased chlorophyll accumulation and a faster rate of photosynthesis.Similar effects were observed in a study by Coppens et al.^59^, where the treatment of tomato plants with dry biomass of *Nannochloropsis* spp., *Ulothrix* spp., and *Klebsormidium* spp. improved photosynthetic activity and the quality of yield.

Bio-stimulants may improve growth through different mechanisms including antioxidant capacity enhancement for more control in ROS production and content in plants. The decrease in H_2_O_2_ levels was attributed to the activation of hydrogen peroxide metabolizing enzyme^60^.The findings suggest that the presence of these microalgal extracts at low concentrations increases the efficiency of redox processes by reducing H_2_O_2_ generation. Similarly, the foliar application of *S. platensis* at 1% significantly decreased the level of H_2_O_2_, MDA and electrolyte leakage in the leaves of rosemary plants, both under normal conditions and in the presence of heavy metal stress, compared to untreated control plants^61^.

*Arthrospira* extract at 2% level resulted in increase in all measured stress markers, suggesting that the metabolic balance of free radicals within the cells of the plant was disrupted. This disruption promoted the generation of free oxygen radicals, leading to oxidative stress^62^, The presence of ROS can accelerate the peroxidation of membrane lipids, which in turn affects cell membrane fluidity and permeability due to changes in the lipid composition^63^.

Foliar spraying with *Chlorella*,* Nannochloropsis,* and *Arthrospira* extracts, particularly at a concentration of 0.5%, significantly increased the levels of non-enzymatic antioxidants [such as total antioxidant capacity, total phenols, and flavonoids] in common bean compared to untreated plants.This agree with **Battacharyya et al.**^52^ who noted that using algae-based extracts can enhance the biosynthesis of plant defense compounds like flavonoids and phenylpropanoid, thereby affecting primary or secondary metabolism. Similarly, treatments with* A. platensis* resulted in increased total phenols, improved growth in onion plants^61^, and elevated total antioxidant capacity, total phenols and flavonoids in rosemary plants at a concentration of 0.2%^61^.

High concentrations of algal extracts showed the lowest phenols and flavonoids content This might be due to inhibiting the biosynthetic pathway of plant defense compounds such as inhibition of phenylalanine ammonia lyase [PAL]. PALresults in the up-regulation of the phenylpropanoid pathway and the production of phenolic, and phytoalexins substances^64^.

Increased activity of antioxidant enzymes is linked to the up-regulation of specific biosynthetic pathways, which promote the generation of secondary metabolites with antioxidant properties. According to **Bulgari et al.**^65^ bio-stimulants improve the overall performance of higher plants, and promoting their growth. They can also encourage the buildup of antioxidant compounds, thus increasing the plants’ tolerance to stress conditions. In this connection, the activity of POX, polyphenol oxidase [PPO], APX, and PAL increased in shoots and roots of rice plants by inoculation with* C. elenkinii*^66^, and tomato [*Solanum lycopersicum*] plants after 48 h injecting with crude polysaccharides [0.2 mg mL^−1^] extract from *C. vulgaris*, *C. reinhardtii*, and *C. sorokiniana*^67^. The activity of CAT and APOX increased first up to 1.0% level of three algal extracts, and then decreased when the concentrations of algal extracts were greater than 1.0%, This reduction in activity may be attributable to the inhibition of CAT and APOX enzymes synthesis or the change in assembly of enzyme subunits at an extremely high concentration of algal extracts.

Improvement of vegetative growth provided plants by sufficient primary metabolites needed during fruiting stage. This improvement of yield may be largely attributed to enrichment of the extracts with nutrients, stimulating biochemical processes in plants, and consequently by boosting the translocation and concentration of certain metabolites in plant organs leading to accumulating dry matter, a greater number of pods per plant, seeds per pod, and pod weight are produced, which are the major parameters in assessing the yield of bean. According to **Dreakeiwicz**^68^ high levels of gibberellic acid in cyanobacteria may promote plant growth by inhibiting chlorophyllase activity and thus promot plant growth. Also, their ability to stimulate endogenous hormone synthesis in the treated plants^69^. Cyanobacteria produce more endogenous and exogenous auxins in the presence of wheat plant, indicating that plants may release signals responsible for higher auxin production^70^. *Chlorella* genus provides with high amounts of growth promoting factors, like cytokinins identified as iso-pentenyl adenine, zeatin, and its conjugated ribosides^34^. Some microalgae liquid extracts have proved to trigger biochemical processes that lead to accumulation of vital metabolites resulting in improvement of qualitative traits of the final marketable products^49^. In accordance, foliar spraying with* S. platensis* aqueous extract at 3% increased the fresh biomass of aerial parts in tomato plants by 48%, the plant length by 19%, the diameter by 33%, and the fruit biomass by 43%^71^.

Furthermore, the present report reveals better performances in common bean yield criteria. Our findings confirmed the observations of Dineshkumar et al.^25^ and Refaay et al.^72^ who noted that *Chlorella vulgaris*, singly, or combined with chemical fertilizer improved yield characters including number of pods plant^−1^, number of seed plant^−1^, and pods dry weight in black gram [*Vigna mungo* [L.] and common beans plants.

Suppression in plant yield at 2.0% level may be linked to increase chlorophyllase activity due to low amounts of gibberellic acid in plants and consequently reduction in chlorophyll content, or the decrease of nutrient absorbed as observed in the present study. This agree with Godlewska et al.^57^ who reported lower content of B, Cu, Fe, Mn, Ni, and Zn in radish following the application of higher concentrations of *A. platensis* growth medium and using foliar fertiliser with Spirufertfi [Tamanduá, Brasil] containing high concentrations [45 g L^−1^] of *Arthrospira* spp. reduced fruit yield and lower pulp firmness in eggplant [*Solanum melongena*]^10^.

The major findings of the study were as follows: moisture ranged from 10.33 to 12.25%, total ash 0.69–1.79%, crude fat 0.56–4.31%, crude fiber 4.62–11.05%, crude protein 23.88–38.31%, total carbohydates 47.57–62.62%, and the range of energy level of the seed flour was [351.04–370.35 kcal g^−1^]. In this respect, the principal components of the Cultivar 112 [*Phaseolus vulgaris* L.] sample per 100 g dry weight, according to **Sahasakul et al.** ^73^ were carbohydrate [70.48 g], protein [23.00 g], fat [1.38 g], ash content [5.13 g], dietary fiber [20.93 g], and energy [386.39 kcal g^−1^]. The values obtained were identical to those discovered in this study, with the exception of the ash content being higher and dietary fiber being significantly lower when compared to our investigation. Cultivars, localities, weather and climate, moisture content, and analysis techniques could all be contributing factors to this variation.

Application of *Chlorella*,* Nannochloropsis,* and *Arthrospira* extracts up to 1.0% level significantly increased the percentage of crude protein , total fat , crude fiber, and ash, as well as energy, while decreased moisture content and total carbohydrates in common bean seeds compared with their respective controls. Similarly, the highest significant increase in total protein and total carbohydrate in *P. vulgaris* seeds were obtained by using *C. vulgaris* suspensions at 10% + chemical fertilizer compared with *A. platensis*, and *Tetradesmus dimorphus* treatments^72^. Also, foliar application of *C. vulgaris* extract at 1 mg C_org_ L^−1^ concentration increased protein, and ash contents at the shoot and root levels of lettuce seedlings^55^. In this study, *Nannochloropsis* at 0.5% had the highest values of crude proteins, and ash, as well as high fat , crude fibers , and energy with the lowest total carbohydrates, and moisture content**.** The total protein mean content of common bean treatments were in the range of 23.88–38.31%, the lowest and highest values obtained by* S. platensis* at 2.0% and* N. salina* at 0.50%, respectively which was greater than the range of 18.62% [Gobirasha] to 25.98% [Tinike] for Ethiopian common bean varieties^74^*.* The highest value of total ash in this study was recorded by *N. salina* treatment at 0.5% [1.79%], while the lowest value was obtained by *S. platensis* treatment at 2% [0.69%]. The level of ash is corresponds to the quantity of minerals in the food. Our results were lower than the findings of Jepleting et al.^75^ who reported a total ash content range of 3.98- 4.12% for two improved Kenyan bean varieties. Crude fat provides a very good source of energy and has a great role in transport of fat soluble vitamins, insulates, protects internal tissues and contributes to important cells processes ^76^. Based on this study, *C. vulgaris* treatment at 0.25% had the highest crude fat among the others with the value of 4.31%, while *S. platensis* treatment at 2.0% was found to be the lowest in crude fat content [0.56%]. The present results of lipid content of most common bean treatments were higher than range of 0.84% [Sari-I] to 2.86% [Hundane] reported by Ketema et al.^74^ for Ethiopian common bean varieties. In this study, moisture content of *N. salina* at 0.5%—treated plants was found to be the lowest with the mean value of [10.33%] and was significantly different from the highest value of 12.25% with *S. platensis* at 2.0% treatment. In this context, the application of both the *A. platensis* extract alone and the mixture at two different fertilization levels significantly improved bulb quality and conservation, as the treated plants had larger bulb diameters and total soluble solids contents and less cumulative weight loss during storage ^26^. The low moisture content of dried beans facilitates their transportation, storability and prolongs their shelf life^77^, while, beans with a moisture content over 13% considerably lose flavour and texture within six months ^78^. The values obtained in this study were comparable with other studies done for common bean varieties ^79^. However, the values were lower than [13.89–15.62%] those reported by Brigide et al.^80^ for biofortified bean varieties grown in Brazil, and higher than [7.85–10.69] those reported by Jepleting et al.^75^ for two improved bean varieties in Kenya. The present study demonstrated that *C. vulgaris* at 0.25% tended to exhibit the highest dietary fiber and also exhibited the highest TF [4.31%], and high protein [31.94%] content, leading to the highest food energy**.** Beans are high-fiber food that helps in various physiological effects for human health. Crude fiber is known to influence production of high butyrate levels and butyrate has been linked to lower risks for cancer ^81^. In this research, the crude fiber content of common bean had the highest value of 11.05% with* C. vulgaris* at 0.25%, while the lowest value of 4.62% with* S. platensis* treatment at 2.0%. These results were higher than crude fiber content range of 3.31 and 4.31% for Faida [biofortified] and RM 01 [drought tolerant] Kenyan bean varieties, respectively as demonstrated by Jepleting et al.^75^. The highest food energy level of the seed was recorded by ***C. vulgaris*** at 0.25% [370.35 kcal g^−1^], whereas the lowest energy mean value was obtained by *S. platensis* at 2.0% [351.04 kcal g^−1^]. These values were lower than the range of energy contribution [386.07 – 489.78 kcal 100 g^−1^ DW] of 10 bean cultivars reported by Sahasakul et al.^73^. The results obtained indicate that *S. platensis* at 2.0% had the highest value of TC [62.62%] in seeds, but the lowest level of ash, and CF. Also, the lowest level of CP, and TF leading to the lowest energy [351.04 kcal g^−1^]. Carbohydrates are major components of dry beans that have a low glycemic index which is considered a therapeutic diet for diabetes patients ^79^. In this study, *N. salina* at 0.5% had the lowest total carbohydrate content with the mean value of [47.57%]. In accordance, Ketema et al.^74^ reported that the carbohydrate content observed was within the range of 58.21% [hundane] to 66.36% [Gobirasha] for 23 Ethiopian common bean varieties.

*Nannochloropsis* treated plants at 0.5% has the highest N, P, K, Mg and Ca content in seed flour followed by *Chlorella* at 0.5% concentration. In accordance, microalgae offers substantial levels of macro and micronutrients, metabolites, and can increase nutrient uptake in plants^53^. Foliar applied *A. platensis* hydrolysate enhanced the content of nitrogen, phosphorus, potassium, magnesium and calcium in petunia at 10 gL^−1^^21^, and was superior in increasing protein content, phosphorus, and potassium uptake in spinach leaves than soil application of an *Anabaena sphaerica* aqueous extract at similar concentration^82^. *C. vulgaris* mixed with *A. platensis* improved the availability of nitrogen, phosphorus and potassium in rice plants^54^, and improved the proximate composition by increasing sodium, potassium, calcium, magnesium and phosphorus contents of black gram seed flour at 4.5 and 5.0 ml *C. vulgaris* cell extracts^25^.

Foliar application of *Arthrospira* spp. at 10 g L^−1^ did not affect nitrogen, phosphorus, potassium, and sodium content in aubergine [*Solanum melongena*] leaf^12^, while decreased the concentrations of Fe, Mn, Zn, B, Cu, and Ni in radish at concentrations higher than 20% ^57^.

## Conclusions

Our research reveals that the application of *C. vulgaris*, *A. platensis,* and* N*. *salina* extracts up to 1.0% promoted common bean [*Phaseolus vulgaris*] growth, chlorophyll content index, yield attributes, and quality of yielded seeds, among which 0.5% concentrations provided better results. These three microalgal extracts alleviated membrane lipid peroxidation and prevented the generation and accumulation of ROS via adjusting the activity of enzymatic and non-enzymatic antioxidant defence systems in common bean plants relative to controls.

*N*. *salina* at a concentration of 0.5% is the most effective and ideal bio-stimulant to improve plant growth and yield quality by maximising gains in crude proteins, ash, and minerals, as well as increasing fat, crude fibres, and energy, along with the lowest moisture percent in seed flour. In contrast, higher concentrations of microalgal extract had a suppressive impact on yield and growth.

Consequently, this research opens the possibility of utilising foliar spray with *C. vulgaris*, *S. platensis,* and* N*. *salina* extracts up to 1.0% as an effective bio-stimulant, safe, eco-friendly, and easy implementable remedy for world-wide sustainable agriculture.

## Data Availability

All data generated or analyzed during this study are included in this article.
